# Individualization of PEEP and tidal volume in ARDS patients with electrical impedance tomography: a pilot feasibility study

**DOI:** 10.1186/s13613-021-00877-7

**Published:** 2021-06-02

**Authors:** Tobias Becher, Valerie Buchholz, Daniel Hassel, Timo Meinel, Dirk Schädler, Inéz Frerichs, Norbert Weiler

**Affiliations:** Department of Anesthesiology and Intensive Care Medicine, University Medical Center Schleswig-Holstein, Campus Kiel, Kiel, Germany

**Keywords:** Electrical impedance tomography, Lung-protective ventilation, Ventilator-induced lung injury, Personalized medicine, Acute respiratory failure

## Abstract

**Background:**

In mechanically ventilated patients with acute respiratory distress syndrome (ARDS), electrical impedance tomography (EIT) provides information on alveolar cycling and overdistension as well as assessment of recruitability at the bedside. We developed a protocol for individualization of positive end-expiratory pressure (PEEP) and tidal volume (*V*_T_) utilizing EIT-derived information on recruitability, overdistension and alveolar cycling. The aim of this study was to assess whether the EIT-based protocol allows individualization of ventilator settings without causing lung overdistension, and to evaluate its effects on respiratory system compliance, oxygenation and alveolar cycling.

**Methods:**

20 patients with ARDS were included. Initially, patients were ventilated according to the recommendations of the ARDS Network with a *V*_T_ of 6 ml per kg predicted body weight and PEEP adjusted according to the lower PEEP/FiO_2_ table. Subsequently, ventilator settings were adjusted according to the EIT-based protocol once every 30 min for a duration of 4 h. To assess global overdistension, we determined whether lung stress and strain remained below 27 mbar and 2.0, respectively.

**Results:**

Prospective optimization of mechanical ventilation with EIT led to higher PEEP levels (16.5 [14–18] mbar vs. 10 [8–10] mbar before optimization; *p* = 0.0001) and similar *V*_T_ (5.7 ± 0.92 ml/kg vs. 5.8 ± 0.47 ml/kg before optimization; *p* = 0.96). Global lung stress remained below 27 mbar in all patients and global strain below 2.0 in 19 out of 20 patients. Compliance remained similar, while oxygenation was significantly improved and alveolar cycling was reduced after EIT-based optimization.

**Conclusions:**

Adjustment of PEEP and *V*_T_ using the EIT-based protocol led to individualization of ventilator settings with improved oxygenation and reduced alveolar cycling without promoting global overdistension.

*Trial registration*This study was registered at clinicaltrials.gov (NCT02703012) on March 9, 2016 before including the first patient.

**Supplementary Information:**

The online version contains supplementary material available at 10.1186/s13613-021-00877-7.

## Background

Mechanical ventilation is a life-saving treatment for critically ill patients suffering from acute respiratory distress syndrome (ARDS). The morphological features of ARDS, namely regional atelectasis, overdistension and presence of lung inhomogeneities pose patients at an increased risk of developing ventilator-induced lung injury (VILI). Cyclic opening and closing of lung units may lead to atelectrauma, whereas ventilation at high lung volumes may lead to overdistension and barotrauma [1]. The potentially detrimental effects of ventilation at high absolute lung volumes can be quantified using the concept of stress and strain: when the lungs are inflated with elastance-based transpulmonary pressure (stress) of 27 mbar, they typically reach a strain of 2.0, corresponding to an inflation to twice their resting volume (functional residual capacity, FRC) [2]. Conceivably, any further inflation to even higher volumes increases the risk of VILI development because of global overinflation [3]. Regionally, detrimental levels of stress may be reached at even lower values of global transpulmonary pressure due to the presence of regional inhomogeneities which act as local pressure multipliers (“stress raisers”, [4]).

In theory, the negative effects of both overdistension and alveolar cycling could be counterbalanced by adjusting positive end-expiratory pressure (PEEP) according to global respiratory system compliance (*C*_rs_), which would then lead to ventilation with minimized airway driving pressure (Δ*P*_aw_) and presumably less harm to the lungs [5]. However, PEEP titration according to global *C*_rs_ has failed to show beneficial results in a large multi-center trial [6]. This could, in part, be explained by the fact that changes in global *C*_rs_ with PEEP are a weak predictor of recruitability [7]. Global *C*_rs_ primarily reflects changes in the mechanical properties of lung tissue already open for ventilation; atelectasis formation and reopening are comparatively slow processes that may take some time to translate into global changes in *C*_rs_ [8].

Electrical impedance tomography (EIT) allows bed-side assessment of regional changes in *C*_rs_. This information can be used to identify cyclic opening and closing of lung units (9) as well as regional overdistension [9, 10] and for early detection of even small changes in lung recruitment [10]. EIT-derived regional ventilation delay inhomogeneity (standard deviation of regional ventilation delay, SD_RVD_) is closely correlated to alveolar cycling as assessed by end-expiratory and end-inspiratory computed tomography (CT) scans [11]. A ventilator protocol incorporating EIT-derived information could be used to adjust ventilator settings in a way that avoids both alveolar cycling and regional overdistension, thus reducing the risk for VILI. Despite this potential, reports on prospective optimization of mechanical ventilation with EIT are scarce. Previous studies using EIT to guide mechanical ventilation have focused primarily on adjustment of PEEP [12, 13] without individual adjustment of tidal volumes (*V*_T_). Here, we describe a protocol for individual optimization of both PEEP and* V*_T_ with EIT and we report its effects on global lung stress and strain, oxygenation, lung recruitment, SD_RVD_ and other physiologic variables in a pilot feasibility study including 20 mechanically ventilated intensive care unit (ICU) patients.

## Methods

We conducted a pilot feasibility study (clinicaltrials.gov NCT02703012) including 20 adult ICU patients ventilated in pressure-controlled mode with no spontaneous breathing activity. All patients had ARDS according to the Berlin Definition [14]. Exclusion criteria were severe hemodynamic instability, thoracic skin lesions, pregnancy, severe chronic obstructive pulmonary disease, esophageal pathologies, presence of cardiac pacemaker, duration of ARDS more than 72 h and inspired oxygen fraction (FiO_2_) of more than 80%. Informed consent was obtained from the patients’ legal representatives.

### Measurements

The EIT device (PulmoVista 500, Dräger, Lübeck, Germany) was connected to the ventilator (Evita XL or V500, Dräger). Synchronized ventilator and EIT data were recorded at sampling rates of 50 Hz. Hemodynamic data, air flow, airway pressure (*P*_aw_), esophageal pressure as well as inspired and expired O_2_ and CO_2_ were additionally recorded with an S/5 monitoring system (Datex-Ohmeda, Helsinki, Finland) and stored electronically. The validity of esophageal pressure measurements was confirmed using an expiratory hold maneuver with gentle manual chest compressions. Cardiac output was assessed by transpulmonary thermodilution (PiCCO, Pulsion, München, Germany), where available.

### Study procedure

Adjustment of ventilator settings according to the ARDS Network protocol and according to the EIT protocol was performed in sequential order without randomization. During the first 2 h of measurement, *V*_T_, respiratory rate (RR) and PEEP were adjusted according to the recommendations of the ARDS Network protocol with *V*_T_ of 6 ml/kg predicted body weight (PBW) and PEEP setting according to the lower PEEP/FiO_2_ table of the ARMA trial [15]. Subsequently, an arterial blood gas (ABG) sample was taken and the first assessment of SD_RVD_, stress and strain was performed. Ventilator settings were then optimized according to the EIT-based protocol once every 30 min for a total of 4 h. At the end of the 4 h period, another assessment of SD_RVD_, stress and strain was performed.

### EIT protocol

Recruitability was assessed using a sustained-inflation maneuver with *P*_aw_ of 40 mbar applied for a duration of 40 s or until a decrease in systolic arterial pressure by more than 20% was observed, followed by a PEEP increase of 3 mbar.

Regional *C*_rs_ was assessed by dividing the EIT image in four horizontal regions of interest (ROIs) and by multiplying global *C*_rs_ with the relative tidal impedance change in each of the ROIs. For assessment of recruitability, we analyzed changes in regional *C*_rs_ occurring after a sustained-inflation maneuver with *P*_aw_ of 40 mbar followed by a PEEP increase of 3 mbar. If, in any of the four ROIs, a regional increase in *C*_rs_ by more than 3% (normalized to global *C*_rs_) was identified following the sustained-inflation maneuver and PEEP increase, the recruitment maneuver was classified as “successful” and the higher PEEP level was kept. Alveolar cycling and overdistension were analyzed by halving inspiratory driving pressure (Δ*P*) for diagnostic purposes for about three consecutive breaths. If a reduction in regional *C*_rs_ by more than 3% (normalized to global *C*_rs_) was observed in any of the four ROIs during ventilation with lower Δ*P*, this was interpreted as alveolar cycling, and PEEP was increased by 3 mbar. An increase in regional *C*_rs_ in any ROI by more than 3% (normalized to global *C*_rs_) with lower Δ*P* was interpreted as overdistension.

**Fig. 1:**
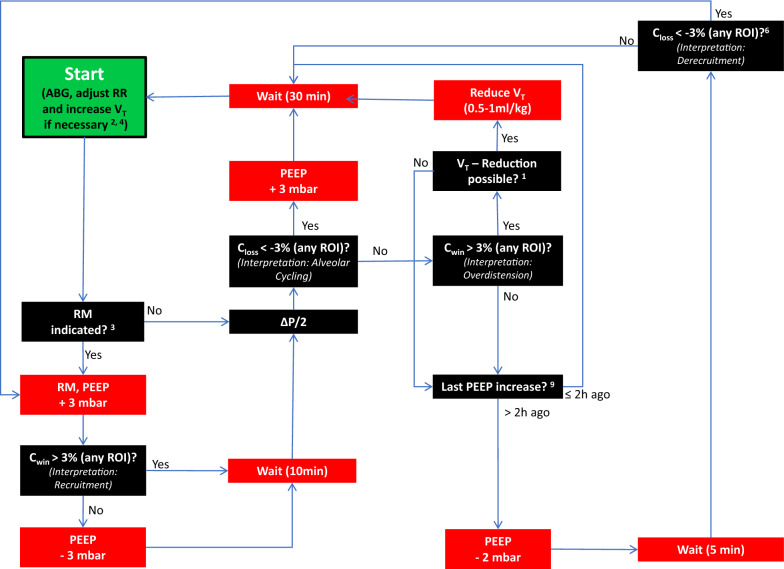
Clinical protocol for prospective optimization of ventilator settings with electrical impedance tomography (EIT). Optimization started with an arterial blood gas (ABG) analysis and, if necessary, adjustments of respiratory rate (RR) and tidal volume (V_T_), followed by a recruitment maneuver (RM) and subsequent adaptations of positive end-expiratory pressure (PEEP) and V_T_. The footnotes are explained in the Additional file 1

In this case, V_T_ was decreased by 1 ml/kg PBW provided this did not lead to severe acidosis (pH < 7.2). PEEP was decreased by 2 mbar if no recruitability and no alveolar cycling had been identified during the last 2 h. The details of the EIT protocol are presented in Fig. 1 and in the Additional file 1.

### Assessment of ventilation delay, stress and strain

Starting at the set PEEP level, a low-flow pressure–volume maneuver with an inspiratory flow of 6 l/min and an inspiratory *V*_T_ of 12 ml per kg PBW was performed to allow assessment of SD_RVD_ as described by Muders and coworkers [11]. SD_RVD_ was calculated offline by analyzing the EIT data obtained during the low-flow pressure–volume maneuver with the “Diagnostics” view of the PC version of PulmoVista 500 Software 1.2 (Dräger Medical, Lübeck, Germany).

Subsequently, FiO_2_ was increased by 10% and decreased to its original value after 10 min to allow calculation of end-expiratory lung volume (EELV) according to [16]. Total inspiratory lung volume (*V*_insp_) was calculated by adding *V*_T_ to EELV: *V*_insp_ = EELV + *V*_T_.

For assessment of FRC, we performed an expiratory release maneuver by setting PEEP to zero and allowing complete exhalation of inspired air to ambient pressure for a duration of 10 s. Expired volume during this maneuver (release volume, *V*_release_) constitutes the difference between *V*_insp_ and the relaxation volume of the respiratory system. It was then used to calculate release-derived FRC (FRC_release_) by subtracting *V*_release_ from *V*_insp_: FRC_release_ = *V*_insp_–*V*_release_. Subsequently, global lung strain was calculated as the ratio of *V*_release_ to FRC: Strain_release_ = *V*_release_/FRC.

This approach may lead to an underestimation of actual FRC because of alveolar derecruitment that may occur during complete exhalation to ambient pressure. Therefore, we additionally calculated recruitment-adjusted FRC (FRC_recr_) by first calculating the assumed PEEP volume (*V*_PEEP_) by multiplying PEEP with global *C*_rs_ (*V*_PEEP_ = *C*_rs_ * PEEP) and subsequently subtracting *V*_PEEP_ from EELV: FRC_recr_ = EELV–*V*_PEEP._

Recruitment-adjusted strain (strain_recr_) was then calculated as the ratio of end-inspiratory lung volume to FRC_recr_: Strain_recr_ = *V*_insp_/FRC_recr_.

For assessment of airway plateau pressure and transpulmonary plateau pressure (*P*_aw,plat_;* P*_tp,plat_), we performed an end-inspiratory airway occlusion of 3–4 s. Airway driving pressure (Δ*P*_aw_) was calculated as the difference between *P*_aw,plat_ and PEEP: Δ*P*_aw_ = *P*_aw,plat_–PEEP. Total end-expiratory transpulmonary pressure (*P*_tp,exp_) was calculated as the difference between PEEP and end-expiratory esophageal pressure and transpulmonary driving pressure (Δ*P*_TP_) was calculated as difference between *P*_tp,plat_ and *P*_tp,exp_: Δ*P*_TP_ = *P*_tp,plat_—*P*_tp,exp_. Respiratory system elastance (*E*_rs_) and lung elastance (*E*_lung_) were calculated from the ratio of Δ*P*_aw_ and Δ*P*_TP_ to expired *V*_T_. Stress was calculated from P_aw,plat_ multiplied with the ratio between *E*_lung_ and *E*_rs_: Stress = *P*_aw,plat_ * *E*_lung_/*E*_rs_.

Specific lung elastance (*E*_lung,spec_) was calculated as the ratio between end-inspiratory stress and strain_release_: *E*_lung,spec_ = Stress/Strain_release_.

Tidal power was calculated as described by van der Staay and Chatburn [17], describing inspiratory mechanical power without the resistive portion and the energy which escapes to atmosphere during expiration: tidal power = 0.098 * RR * Δ*P*_aw_ * *V*_T_/2 (with RR = respiratory rate per minute; *V*_T_ = tidal volume in litres, Δ*P*_aw_ = airway driving pressure in mbar).

Of note, ventilation delay, stress and strain were not used to optimize ventilator settings but as physiological endpoints only.

### End points and statistical analysis

The primary end point was the number of patients with stress below 27 mbar and release-derived strain below 2.0 after 4 h of ventilation according to the EIT-based protocol. Secondary endpoints included changes in SD_RVD_, *C*_rs_, Δ*P*_aw_ and PaO_2_/FiO_2_. As exploratory endpoints, we analyzed changes in lung compliance (*C*_*lung*_), Δ*P*_TP_, *P*_tp,exp_, tidal power, recruitment-adjusted strain and cardiac output, where available. Statistical analysis was performed with GraphPad Prism 5.0 (GraphPad, LaJolla, USA). Normal distribution was assessed with Shapiro–Wilk test. Continuous variables are presented as mean ± standard deviation if normally distributed or as median [interquartile range, IQR] if not normally distributed. Comparisons were performed with two-sided paired *t* test or Wilcoxon matched-pairs test, as appropriate.

## Results

20 patients (11 male, 9 female; age 65 ± 15 years, height 172 ± 9 cm, weight 77 ± 20 kg) were included. One patient had mild ARDS, 18 patients presented with moderate ARDS and one patient fulfilled the criteria for severe ARDS. The average duration of mechanical ventilation prior to study inclusion was 47 ± 18 h. 14 patients had cardiac output measurements using the PiCCO device. Baseline patient characteristics are presented in Table 1.

**Table 1:** Main characteristics of the study population

Patient No.	Age (years)	Sex (M/F)	BMI (kg/m^2^)	PaO_2_/FiO_2_ (mmHg)	ARDS Type (pulmonary/extrapulmonary)	Hours of MV before enrollment	PEEP (mbar)	28 day survivor?
1	65	F	21	137	Pulm	31	10	No
2	72	F	29	148	Extrapulm	61	8	Yes
3	80	M	29	120	Extrapulm	32	8	Yes
4	44	F	23	132	Extrapulm	43	7	Yes
5	78	F	24	148	Extrapulm	34	10	Yes
6	28	M	32	189	Pulm	33	8	Yes
7	71	M	26	162	Pulm	30	10	Yes
8	54	M	16	215	Pulm	30	8	Yes
9	78	M	25	168	Pulm	55	8	Yes
10	53	M	22	152	Pulm	62	10	No
11	69	M	28	194	Extrapulm	72	10	Yes
12	59	F	26	135	Pulm	54	12	No
13	76	M	27	138	Pulm	70	8	Yes
14	86	M	25	138	Pulm	42	8	No
15	70	F	27	182	Extrapulm	18	8	Yes
16	74	F	23	190	Puml	35	10	No
17	73	F	24	127	Extrapulm	72	10	No
18	49	M	40	105	Extrapulm	62	10	Yes
19	50	F	21	145	Pulm	72	10	Yes
20	73	M	29	96	Pulm	24	18	No
Mean (± SD)	65 (± 15)	11 M, 9 F	26 (± 5)	151 (± 31)	12 Pulm., 8 extrapulm	47 (± 18)	10 (± 2)	13 survivors

*M* male, *F* female, *BMI* body mass index, *PaO*_*2*_*/FiO*_*2*_ ratio of arterial partial pressure of oxygen to inspired fraction of oxygen, *ARDS* acute respiratory distress syndrome, *pulm.* Pulmonary, *extrapulm*. Extrapulmonary, *MV* mechanical ventilation, *PEEP* positive end-expiratory pressure

After adjusting mechanical ventilation according to the ARDS Network protocol, patients were ventilated with a median PEEP level of 10 [IQR 8–10] mbar and an expiratory *V*_T_ of 5.8 ± 0.5 ml/kg PBW. *P*_aw,plat_ was 20.3 [IQR 18.5–22.4] mbar resulting in a Δ*P*_aw_ of 10.4 ± 2.2 mbar and *C*_rs_ of 38.2 ± 8.8 ml/mbar. PaO_2_/FiO_2_ was 151 ± 31 mmHg. Lung stress was within the physiological range for all patients (14.1 ± 3.9 mbar). EELV was 1637 [IQR 1450–2228] ml, corresponding to a release-derived FRC of 1176 ± 439 ml and recruitment-adjusted FRC of 1267 [IQR 1141–1803] ml. Release-derived strain was above 2.0 for 2 patients (2.2 and 3.4, respectively) with a median value of 0.80 [IQR 0.70–1.10]. Recruitment-adjusted strain was below 2.0 for all patients with an average value of 0.56 ± 0.14. The ventilation-delay inhomogeneity SD_RVD_ was 8.3 ± 2.8%.

During the first assessment of recruitability with EIT, we found recruitable lung tissue in 16 patients. The first assessment of overdistension and alveolar cycling with EIT revealed regional overdistension in 15 patients and alveolar cycling in 5 patients. A median of 3 [IQR 3–4] assessments of recruitability, 6 [IQR 5–6] assessments of overdistension and 6 [IQR 5–6] assessments of alveolar cycling were performed over the 4-h period of optimization of ventilator settings according to the EIT-based protocol, resulting in 3 [IQR 3–4] adjustments of PEEP and 2 [IQR 1–2] adjustments of * V*_T_. The individual treatment courses of all patients during EIT-based adjustment of ventilator settings are presented in the Additional file 1: (pages 5–9). One patient example illustrating all steps of the protocol with EIT screenshots is presented in the Additional file 1: (pages 16–25).

**Fig. 2:**
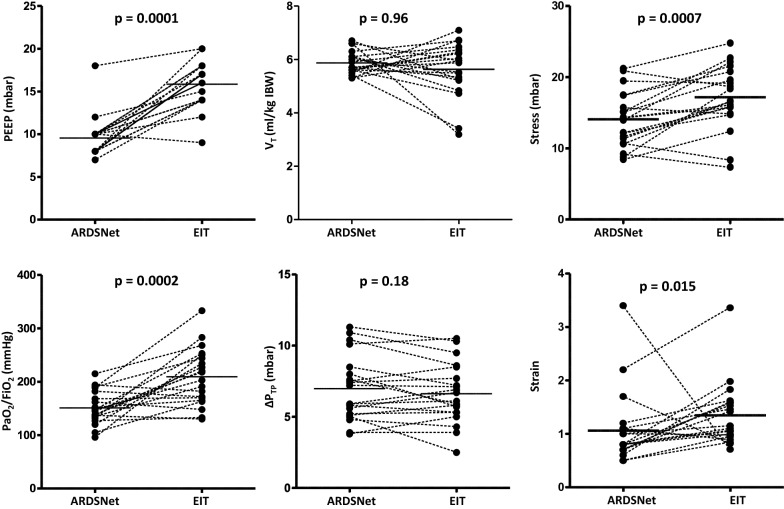
Changes in positive end-expiratory pressure (PEEP), tidal volume (*V*_T_), elastance-based end-inspiratory transpulmonary pressure (Stress), ratio of arterial partial pressure of oxygen to inspired fraction of oxygen (PaO_2_/FiO_2_), transpulmonary driving pressure (Δ*P*_TP_) and strain after individualization of ventilator settings according to the electrical impedance tomography (EIT) protocol. *ARDSNet* = Ventilator settings according to acute respiratory distress syndrome network recommendations with low PEEP/FiO_2_ table; EIT = ventilator settings after 4 h of adjustment according to EIT-based protocol

At the end of optimization of ventilator settings according to the EIT-based protocol, EIT identified regional overdistension in 10 patients and alveolar cycling in 0 patients. The set PEEP level had increased to 16.5 [IQR 14–18] mbar (*p* = 0.0001), while the average expiratory* V*_T_ remained similar, though with higher intra-individual variability (5.7 ± 0.9 ml/kg PBW; *p* = 0.96; Fig. 2).* P*_aw,plat_ increased to 27.9 [IQR 25.4–29.1] mbar (*p* = 0.0001), resulting in Δ*P*_aw_ of 10.4 ± 2.0 mbar (*p* = 0.96) and *C*_rs_ of 34.5 ± 10.3 ml/mbar (*p* = 0.55). PaO_2_/FiO_2_ increased to 209 ± 53 mmHg (*p* = 0.0002). Stress increased significantly to 17.2 ± 4.4 mbar (*p* = 0.0007 in comparison to ARDS Network strategy) but remained below 27 mbar in all patients. In one patient, stress was 24.8 mbar, all other patients remained below 24 mbar after adjustment with EIT (individual patient data reported in Additional file 1: Table S1). Similarly, release-derived strain increased significantly to a median value of 1.13 [IQR 0.96–1.59] (*p* = 0.015) and was above 2.0 in one patient (3.4). Recruitment-adjusted strain remained unchanged despite the higher PEEP levels selected with the EIT-based strategy (0.55 ± 0.19; *p* = 0.77). SD_RVD_ decreased significantly to 6.6 ± 1.9% (*p* = 0.02). Tidal power decreased from 4.96 ± 1.87 to 4.24 ± 1.24 J/min (*p* = 0.047). Individual patient values of PEEP, *V*_T_, PaO_2_/FiO_2_ and Δ*P*_TP_ are presented in Fig. 2.

No significant changes were found for vasopressor dose, cardiac output, PaCO_2_ and pH. The results are summarized in Table 2. All individual patient results are presented in the (Additional file 1: Tables S1–S6, pages 10–15).

**Table 2:** Ventilator data and physiological parameters after mechanical ventilation according to the ARDS Network protocol low positive end-expiratory pressure (PEEP) table (ARDSNet) and after 4 h of mechanical ventilation according to the electrical impedance tomography based protocol (EIT Protocol)

Parameter (unit)	ARDSNet	EIT Protocol	*p*
Stress (mbar)	14.1 ± 3.9	17.2 ± 4.4	0.0007
Strain_release_ (ratio)	0.80 [0.70–1.10]	1.13 [0.96–1.59]	0.015
*E*_lung,spec_ (mbar)	15.9 ± 5.8	14.2 ± 4.8	0.26
Strain_recr._ (ratio)	0.56 ± 0.14	0.55 ± 0.19	0.77
PaO_2_/FiO_2_ (mmHg)	151 ± 31	209 ± 53	0.0001
PaCO_2_ (mmHg)	58 ± 11	61 ± 9	0.28
pH	7.31 ± 0.07	7.29 ± 0.05	0.17
*V*_T_ (ml/kg PBW)	5.8 ± 0.47	5.7 ± 0.92	0.96
*P*_aw.plat_ (mbar)	20.3 [18.5–22.4]	27.9 [25.4–29.1]	0.0001
PEEP (mbar)	10 [8–10]	16.5 [14–18]	0.0001
*C*_rs_ (ml/mbar)	38.2 ± 8.8	37.9 ± 11.4	0.83
V_PEEP_ (ml)	348 [311–457]	603 [400–809]	0.0004
*C*_lung_ (ml/mbar)	60.07 ± 23.48	63.53 ± 26.38	0.33
Δ*P*_aw_ (mbar)	10.4 ± 2.2	10.4 ± 2.0	0.96
Δ*P*_TP_ (mbar)	7.0 ± 2.3	6.6 ± 2.1	0.19
SD_RVD_ (%)	8.3 ± 2.8	6.6 ± 1.9	0.022
*P*_tp,plat_ (mbar)	4.9 [1.4–6.9]	17.9 [15.6–19.0]	< 0.0001
*P*_tp,exp_ (mbar)	-3.5 [-5.9–1.1]	3.25 [-0.7–4.7]	0.0002
Tidal power (J/min)	4.96 ± 1.87	4.24 ± 1.24	0.047
EELV (ml)	1637 [1450–2228]	2348 [2034–3201]	< 0.0001
FRC_release_ (ml)	1176 ± 439	1317 ± 443	0.17
FRC_recr._ (ml)	1267 [1141–1803]	1704 [1496–2512]	0.0001
Cardiac Index* (l/min/m^2^)	3.4 [2.4–4.5]*	3.4 [2.5–5.0]*	0.19*
NE (µg/kg/min)	0.12 [0.01–0.26]	0.12 [0.01–0.26]	0.55

Normally distributed variables are presented as mean ± standard deviation, whereas non-normally distributed variables are presented as median [interquartile range]

*PaO*_*2*_ arterial partial pressure of oxygen, *FiO*_*2*_ inspired fraction of oxygen, *C*_rs_ respiratory system compliance, *C*_lung_ lung compliance, *ΔP*_aw_ airway driving pressure, *ΔP*_TP_ transpulmonary driving pressure, *NE* norepinephrine dosage, P_aw.plat_ airway plateau pressure, *PEEP* positive end-expiratory pressure (measured at airway opening), *V*_PEEP_ PEEP volume (calculated by multiplying PEEP with *C*_rs_), *P*_tp,plat_ transpulmonary plateau pressure (calculated as difference between *P*_aw.plat_ and plateau esophageal pressure), *P*_tp,exp_ end-expiratory transpulmonary pressure (calculated as difference between PEEP and end-expiratory esophageal pressure), *SD*_*RVD*_ standard deviation of regional ventilation delay, *EELV* end-expiratory lung volume, *FRC*_*recr*._ recruitment-adjusted functional residual capacity, calculated as EELV–*C*_rs_ * PEEP, *FRC*_*release*_ release-derived functional residual capacity, calculated as EELV–released volume during an exhalation to ambient pressure, *PBW* predicted body weight, *Strain*_*release*_ release-derived strain, calculated as volume change above FRC_rel_, normalized to FRC_rel_, Strain_recr._ recruitment-adjusted strain, calculated as volume change above FRC_recr._, normalized to FRC_recr_, *V*_T_ tidal volume

^*^Cardiac output measurements were available in 14 patients

## Discussion

The main finding of this pilot feasibility study was that adjustment of PEEP and *V*_T_ according to the EIT-based protocol led to individualized ventilator settings with improved oxygenation and lower values of SD_RVD_ consistent with a reduction in alveolar cycling without causing excessive lung stress and strain. Global lung stress remained below 27 mbar in all patients, while release-derived strain was below 2.0 in 19 out of 20 patients. The average value of absolute *P*_TP,exp_ was negative before EIT-based optimization but became positive after optimization. No significant changes in cardiac output and vasopressor dosing were detected, indicating absence of relevant hemodynamic compromise despite the average increase in PEEP levels selected with EIT.

We chose 27 mbar and 2.0 as thresholds for stress and strain, because these values represent the upper limit of the physiological range postulated for human patients in previous publications [2, 18]. In one patient, we found an unphysiologically high value of release-derived strain of 3.4 after adjusting mechanical ventilation with the EIT-based protocol. However, this patient had a moderate *P*_aw,plat_ of 29 mbar and lung stress of 21 mbar. Recruitment-adjusted strain amounted to 1.5, which is still within the physiological range. These findings support the assumption that the high strain observed in this patient was largely due to derecruitment during the release maneuver that was performed for calculating release-derived FRC.

The concept of elastance-based transpulmonary pressure used for calculation of lung stress assumes that at zero airway pressure, transpulmonary pressure also equals zero. This assumption may not always be valid, especially in patients with increased lung weight and ARDS. Elastance-based methods yield different estimates of transpulmonary pressure when compared to the more widely applied method based on absolute values of esophageal pressure [19]. Nonetheless, the assumption of transpulmonary pressure close to zero at zero airway pressure may be an acceptably accurate approximation for the non-dependent lung regions [20]. Therefore, our results may indicate that adjusting PEEP and *V*_T_ with EIT did not lead to overinflation of the non-dependent lung.

Our protocol for prospective optimization of PEEP and *V*_T_ with EIT was largely based on bedside assessment of changes in regional *C*_rs_. An increase in regional *C*_rs_ following a recruitment maneuver was interpreted as indicator for alveolar recruitment and PEEP was increased to keep the recruited lung volume. With this approach, we identified alveolar recruitment in 15 out of 20 patients after the initial recruitment maneuver, indicating recruitability in a large proportion of patients studied.

If regional *C*_rs_ decreased during a brief reduction in* V*_T_, this was interpreted as alveolar cycling and PEEP was increased by a further 3 mbar to counteract this phenomenon. On the opposite, an increase in regional *C*_rs_ during a brief reduction in *V*_T_ was interpreted as overdistension with the previously applied *V*_T_ [9]. In this case, the consequence was to decrease *V*_T_, provided this did not lead to severe acidosis. In 10 out of 20 patients, we still identified regional overdistension with a *V*_T_ of 6 ml/kg PBW which led to further reductions of *V*_T_ to levels below 6 ml/kg PBW. The average V_T_ did not differ after EIT-based optimization, because according to the protocol, *V*_T_ was only decreased when overdistension was detected, while it was also possible to increase it in patients with no detectable overdistension. Of note, changes in PEEP and *V*_T_ did not systematically cause each other: increases in PEEP did not always cause overdistension and force a reduction in *V*_T_. In some instances,* V*_T_ could be maintained or even increased following successful recruitment maneuvers and PEEP increases (see individual patient results reported on Additional file 1: pages 10–15).

Our *C*_rs_-based approach differs from the approach that was employed for prospective optimization of PEEP with EIT in a study published by Eronia and Coworkers [14]. In that study, the time-course of end-expiratory lung impedance (EELI) was analyzed for determining changes in EELV associated with PEEP. A slow decrease in EELI following a recruitment maneuver was interpreted as derecruitment, and PEEP was increased to counteract this phenomenon until a stable EELI was achieved. As EELI appears to be a reasonably accurate measure for changes in EELV [21], this approach allows straightforward bedside assessment of recruitment and derecruitment. However, it is highly susceptible to artifacts: for instance, the pulsation therapy with inflatable mattresses can cause substantial artifacts in EELI [22]; the same applies to changes in torso and arm position [23] and even intravenous fluid therapy, which is a rather common intervention in ICU patients [24, 25]. These interferences render EELI-based analyses of EELV difficult to interpret and error prone. Moreover, while observing EELI may allow bedside assessment of recruitment and derecruitment, it provides no information on regional overdistension, which can be easily identified by analyzing regional changes in *C*_rs_ [9, 10].

Another *C*_rs_-based approach that has been applied for EIT-based optimization of PEEP in patients with ARDS [26] and with COVID-19 induced acute respiratory failure [27, 28] relies on analyzing pixelwise changes in *C*_rs_ during a decremental PEEP trial [10, 29]. This PEEP trial must be started at relatively high PEEP levels (that may be associated with overdistension while applied) and must then be carried on until very low PEEP levels (that may lead to alveolar collapse and atelectasis formation) are reached. Therefore, it cannot be repeated on a regular basis to adapt ventilator settings to the changing conditions of a patient’s lung. In contrast, our approach is primarily based on brief changes in *V*_T_ for diagnosing regional overdistension and alveolar cycling, and can thus be repeated whenever a new assessment of these phenomena is clinically required.

Recent studies have shown that airway closure may occur in up to 40% of patients with ARDS, frequently in conjunction with expiratory flow limitation [30–32]. This could impede the assessment of regional *C*_rs_. A visual inspection of the low-flow pressure–volume loops recorded for assessment of SD_RVD_ as well as of expiratory flow–volume loops recorded during the expiratory release maneuver revealed no evidence of airway collapse or expiratory flow limitation in the patients investigated in this study. Therefore, we cannot draw any direct conclusions regarding the applicability of our approach in patients with airway collapse or expiratory flow limitation. However, if the approach described in this manuscript was executed in a patient with airway collapse, this would most likely lead to lower calculated values of regional *C*_rs_ with reduced* V*_T_. According to the protocol, PEEP would then be increased by 3 mbar, which would, in part, counteract airway collapse. Therefore, we believe that the EIT-based protocol might also be helpful in adjusting PEEP in patients with partial or complete airway collapse.

Our study has several limitations.

1. The study was not randomized. Therefore, we can make no assumptions on whether individualized optimization of mechanical ventilation with our EIT-based protocol has an influence on actual clinical outcomes. The lack of randomization also limits the significance of the results reported in Table 2. Instead, we tried to carefully monitor and describe the physiologic effects of individualized adjustment of ventilator settings with EIT by analyzing changes in transpulmonary pressure and EELV.
2. The thresholds used for defining “physiological” values of stress and strain are somewhat arbitrary, as they are based on animal studies and physiological considerations [2, 3, 18]. Thus, we cannot be certain that global values for stress of 27 mbar and strain of 2.0 are “safe” in all individual patients.
3. This study had a rather complex methodology. However, a simplified protocol focusing only on the EIT measurements that are necessary for the ventilator protocol itself could be applied in future studies. (The measurements of SD_RVD_ or stress and strain are not needed and can be omitted.). This study had a rather complex methodology. However, a simplified protocol focusing only on the EIT measurements that are necessary for the ventilator protocol itself could be applied in future studies. (The measurements of SD_RVD_ or stress and strain are not needed and can be omitted.)
4. This was a single-center pilot feasibility study. Thus, the results of our study cannot readily be generalized to other settings yet.
5. The duration of EIT-based optimization was rather short. The time period of 4 h was selected mainly for practical reasons. For clinical implementation or a future outcome study, the time period of EIT-based optimization needs to be prolonged, with less frequent assessments. Instead of adjusting ventilator settings every 30 min for a total duration of 4 h, a strategy with adjustments performed every 4 h for a total duration of 72 h may be considered (or as needed).
6. No direct measurement of recruitment and alveolar cycling with a reference method, such as CT, was carried out. Our measurements of EELV using the modified nitrogen dilution technique [15] indicated that recruitment-adjusted FRC was significantly increased. The ventilation delay index SD_RVD_, that was not used for optimization of ventilation but served as a secondary outcome parameter, indicated a possible reduction in alveolar cycling with our EIT-based optimization. It should be mentioned that the original approach for calculating regional ventilation delay [11] measures the time from the start of the global impedance–time curve until the regional curve reaches a threshold of 40% of its maximum. In contrast, the algorithm we used for calculating SD_RVD_ in this study compares the time until the regional curve reaches the 40% threshold to the time when the global curve reaches the 40% threshold. Thus, individual pixel RVD values calculated with this approach are negative, when the regional curve precedes the global curve (fast region), but are positive, when the regional curve follows the global curve (delayed region). However, the influence of this difference on the index parameter SD_RVD_, that calculates the standard deviation across all individual pixel values, should be negligible.
7. The low-flow maneuver that was performed for determination of SD_RVD_ may have induced some lung recruitment or at least altered lung volume history. This maneuver was performed both after optimization according to the ARDS Network protocol and after optimization according to the EIT-based protocol. This should have reduced the possible effects of lung recruitment and altered lung volume history on our results and can be expected to further mitigate any possible effect of different calculation methods for SD_RVD_. Nevertheless, independent reference methods, such as CT, might be necessary to confirm a reduction in alveolar cycling following optimization of mechanical ventilation with the EIT-based protocol.
8. The majority of patients included in this study presented with moderate ARDS. Following a meta-analysis by Briel and coworkers [33], recent recommendations suggest using a higher PEEP/FiO_2_ strategy for patients with moderate to severe ARDS [34]. Nevertheless, comparatively low levels of PEEP in patients with moderate to severe ARDS are still common clinical practice in many centers around the world [35].
9. Valid assessment of *C*_rs_, which is a prerequisite for our EIT-based approach for individualization of PEEP and *V*_T_, typically requires a paralyzed patient, even though it is possible to assess *C*_rs_ and Δ*P*_aw_ in many patients on assisted ventilation using an inspiratory-hold maneuver [36, 37]. The patients included in our study exhibited no spontaneous breathing activity. It is uncertain whether a *C*_rs_-based approach using inspiratory hold maneuvers during assisted modes of ventilation will yield similar results in patients with spontaneous breathing activity.

## Conclusions

In conclusion, we presented a protocol for prospective adaption of PEEP and *V*_T_ taking into account EIT-derived information on recruitability, overdistension and alveolar cycling. Mechanical ventilation adjusted according to the EIT-based protocol resulted in global values of lung stress and strain within the physiological limits and was associated with improvements in oxygenation and a reduction in regional ventilation delay inhomogeneity.

## Supplementary Information

**Additional file 1.** Protocol for adjustment of V_T_ and PEEP with EIT, treatment courses of individual patients during EIT-based adjustment, individual patient results and patient example with EIT screenshots

## Data Availability

The data sets used and/or analysed during the current study are available from the corresponding author on reasonable request.

## Abbreviations

ABG: Arterial blood gas
ARDS: Acute respiratory distress syndrome
*C*_rs_: Respiratory system compliance
*C*_lung_: Lung compliance
CT: Computed tomography
Δ*P*: Driving pressure
Δ*P*_aw_: Airway driving pressure
Δ*P*_TP_: Transpulmonary driving pressure
EELV: End-expiratory lung volume
EIT: Electrical impedance tomography
etCO_2_: End-tidal carbon dioxide
FiO_2_: Inspired fraction of oxygen
FRC: Functional residual capacity
FRC_recr_: Recruitment-adjusted functional residual capacity
HR: Heart rate
ICU: Intensive care unit
IQR: Interquartile range
MV: Mechanical ventilation
NE: Norepinephrine
PaCO_2_: Arterial partial pressure of carbon dioxide
PaO_2_: Arterial partial pressure of oxygen
*P*_aw_: Airway pressure
*P*_aw,plat_: Airway plateau pressure
PEEP: Positive end-expiratory pressure
PBW: Predicted body weight
SD_RVD_: Standard deviation of regional ventilation delay
Strain_recr_: Recruitment-adjusted strain
VILI: Ventilator-induced lung injury
*V*_insp_: Total inspiratory lung volume (= EELV + *V*_T_)
*V*_release_: Release volume
*V*_T_: Tidal volume
