# Supplementary material for: Individualization of PEEP and tidal volume in ARDS patients with electrical impedance tomography: a pilot feasibility study
Source: Ann Intensive Care. 2021 Jun 2;11:89. doi: 10.1186/s13613-021-00877-7 (PMC8171998; doi:10.1186/s13613-021-00877-7)

Individualization of PEEP and Tidal Volume in ARDS Patients with Electrical Impedance Tomography – a Pilot Feasibility Study

Tobias H. Becher, Valerie Buchholz, Daniel Hassel, Timo A. Meinel, Dirk Schädler, Inéz Frerichs and Norbert Weiler

Electronic Supplementary Material

Table of Contents

Protocol for adjustment of tidal volume and PEEP with EIT ………………………………………………………………… 2

Treatment courses of individual patients during EIT-based adjustment of ventilator settings ………….. 5

Individual patient results …… 10

Patient Example with EIT Screenshots (Patient 4) ……………………………………………………………………………. 16

Protocol for adjustment of tidal volume and PEEP with EIT

After initial assessment of ventilation delay, stress and strain, ventilator settings were optimized according to the EIT-based algorithm (cf. main manuscript figure 1). Initially, an arterial blood gas (ABG) sample was collected for assessment of arterial partial pressures of oxygen (PaO_2), carbon dioxide ($PaCO_2$) and pH. Respiratory rate (RR) was increased if pH was < 7.30, provided no auto-peep was present. Auto-PEEP was assessed by visual analysis of the flow curve and by performing an end-expiratory occlusion maneuver for direct measurement of air trapping. If auto-PEEP was present or pH >7.40 or arterial partial pressure of carbon dioxide ($PaCO_2$) < 35 mmHg, respiratory rate was decreased.

If RR could not be increased due to the presence of auto-PEEP and pH was below 7.20, V_T was increased by 0.5 to1 ml per kg predicted body weight (PBW).

Recruitablity was assessed using a sustained-inflation recruitment maneuver (RM) with airway pressure (P_{aw}) of 40 mbar followed by a PEEP increase of 3 mbarRegional C_{rs} was assessed by dividing the EIT image in four horizontal regions of interest (ROIs) and by multiplying global C_{rs} with the relative tidal impedance change in each of the ROIs. An increase in regional C_{rs} of more than 5% (normalized to global C_{rs}) was interpreted as recruitment. If recruitable lung tissue was identified following the RM, the higher PEEP level was kept. Tidal recruitment and overdistension were analyzed with a short reduction in V_T that was achieved by halving the inspiratory pressure difference (ΔP) during pressure-controlled-ventilation. Changes in regional C_{rs} during this maneuver were assessed as follows: Regional increases and decreases in C_{rs} were summed up separately and normalized to global C_{rs} with previously set V_T. Any regional increase in C_{rs} with reduced V_T was interpreted as indicative of overdistension with the previously applied V_T, whereas any regional decrease in C_{rs} with reduced V_T was interpreted as indicator for tidal recruitment. If tidal recruitment was identified, PEEP was elevated by 3 mbar. If overdistension was identified, V_T was decreased by 1 ml / kg PBW provided this did not lead to severe acidosis (pH <7.20). PEEP was reduced by 2 mbar if no recruitability and no tidal recruitment had been identified during the last two hours. After any PEEP-reduction, an assessment of derecruiment was performed and the PEEP-reduction was reversed if relevant derecruitment was detected.

The PEEP steps of +3 and -2 mbar were selected pragmatically on the basis of routine clinical practice. We selected different PEEP steps for increase (+3 mbar) and decrease (-2 mbar) to allow the protocol to reach different PEEP levels. If, for example, the "ideal" PEEP for one patient was 12 mbar, and the patient was at that time ventilated with a PEEP of 8 mbar, two consecutive PEEP increases of 3 mbar followed by one PEEP decrease of 2 mbar would have finally resulted in this "ideal" individual PEEP level [8 (+ 3) → 11 (+3) → 14 (-2) → 12].

After four hours of prospective optimization of mechanical ventilation with the EIT-based algorithm, another assessment of ventilation delay, stress and strain was performed for analyzation of primary and secondary outcome measures.

Protocol rules and explanations (cf. main manuscript figure 1)

1) Criteria for V_T reduction:

V_T can be reduced by 0.5 to 1 ml / kg PBW, if

- pH ≥ 7,30 **AND**
- $PaCO_2$ ≤ 75 mmHg **AND**
- current V_T > 4 ml / kg PBW **AND**
- no elevated intracranial pressure

2) Criteria for V_T increase:

V_T must be increased by 0.5 to 1 ml / kg PBW, if

- pH <7,20 **AND**
- RR cannot be increased [4]

  OR
- if current V_T < 8 ml / kg PBW **AND**
- no overdistension **AND**

no V_T – reduction for overdistension during last hour

3) Criteria for recruitment maneuver (RM)

RM shall be performed, if

- PaO_2/FiO_2 < 300 mmHg **AND**
- no unsuccessful RM during last 2 hours **AND**
- no successful RM during last 30 minutes

4) Criteria for adaptation of respiratory rate (RR):

increase RR, if

- pH <7,30 **AND**
- no Auto-PEEP (observe flow curve, perform PEEPi –measurement maneuver if necessary: Auto-PEEP is present if PEEPi > PEEP + 1 mbar)

decrease RR, if

- Auto-PEEP is present (see above) **OR**
- pH ≥ 7,40 **OR**
- $PaCO_2$ < 35 mmHg

5) Recruitment

RM shall be classified as successful, if after 1 min

- regional C_{rs} in any ROI increased by ≥ 3% of global C_{rs} (measured before RM) **OR**
- if global C_{rs} increased by ≥ 10%

in all other cased RM is classified as „unsuccessful"

6) Derecruitment

Derecruitment is present if 5 minutes after a PEEP decrease

- regional C_{rs} in any ROI decreased by > 3% of global C_{rs} (measured before PEEP decrease) **OR**
- global C_{rs} decreased by > 10%

7) Alveolar Cycling

Alveolar Cycling is present, if during a reduction in driving pressure (ΔP) of about 50% for a few breaths,

- C_{rs} at lower ΔP decreases by more than 3% in any ROI (in comparison to global C_{rs} at normal ΔP)

8) Overdistension

Overdistension is present, if during a reduction in ΔP of about 50% for a few breaths

- C_{rs} at lower ΔP increases by more than 3% in any ROI (in comparison to global C_{rs} at normal ΔP)

For diagnosing alveolar cycling and overdistension, a reduction in ΔP by 50% for at least 5 breaths must always be performed.

9) PEEP Changes

Driving pressure (ΔP) must always be kept constant during any changes in PEEP by changing inspiratory pressure by the same value.

„Last PEEP-increase" refers to

- the last successful recruitment maneuver (that is always accompanied by PEEP increase) **OR**
- the last PEEP increase because of „alveolar cycling".

Unsuccessful recruitment maneuvers are not referred to as „Last PEEP increase".

Treatment courses of individual patients during EIT-based adjustment of ventilator settings

Abbreviations: RM = recruitment maneuver; ΔP/2 = reduction in driving pressure (ΔP) of about 50% for a few breaths; CW = compliance win; CL = compliance loss; OD = overdistension; AC = alveolar cycling.

**Pat. 1**	**PEEP (set)**	**Maneuver**	**Finding**	**Interpretation**	**Therapeutic Measure**
1.	10	RM	CW	Recruitment	PEEP increased
2.	13	ΔP/2	CW	OD	VT decreased
3.	16	RM	CW	Recruitment	PEEP increased
4.	16	ΔP/2	CW	OD	No Change (pH < 7.3)
5.	16	RM	No CW or CL	No Recruitment	No Change (pH < 7.3)
6.	16	ΔP/2	CW	OD	No Change (pH < 7.3)
7.	16	ΔP/2	CW	OD	No Change (pH < 7.3)
8.	16	ΔP/2	CW	OD	initiate PEEP decrease
9.	14	PEEP decreased	CW	Less OD, no derecruitment	No Change
10.	14	ΔP/2	CW	OD	initiate PEEP decrease
11.	12	PEEP decreased	CW	Less OD, no derecruitment	No Change
12.	12	ΔP/2	No CW or CL	No relevant OD or AC	No Change

**Pat. 2**	**PEEP (set)**	**Maneuver**	**Finding**	**Interpretation**	**Therapeutic Measure**
1.	8	RM	CW	Recruitment	PEEP increased
2.	11	ΔP/2	No CW or CL	No relevant OD or AC	No Change
3.	11	RM	CW	Recruitment	PEEP increased
4.	14	ΔP/2	No CW or CL	No relevant OD or AC	No Change
5.	14	RM	CW	Recruitment	PEEP increased
6.	17	ΔP/2	No CW or CL	No relevant OD or AC	No Change
7.	17	RM	No CW or CL	No Recruitment	No Change
8.	17	ΔP/2	No CW or CL	No relevant OD or AC	No Change

**Pat. 3**	**PEEP (set)**	**Maneuver**	**Finding**	**Interpretation**	**Therapeutic Measure**
1.	8	RM	No CW or CL	No Recruitment	No Change
2.	8	ΔP/2	CW and CL	OD and AC	PEEP increased, VT decrease[illegible]
3.	11	ΔP/2	CW	OD	VT decreased
4.	11	ΔP/2	CW and CL	OD and AC	PEEP increased, VT decrease[illegible]
5.	14	ΔP/2	CW	OD	VT decreased
6.	14	ΔP/2	No CW or CL	No relevant OD or AC	No Change

Pat. 4	PEEP (set)	Maneuver	Finding	Interpretation	Therapeutic Measure
1.	7	RM	CW	Recruitment	PEEP increased
2.	10	ΔP/2	CL	AC	PEEP increased
3.	13	ΔP/2	CW	OD	VT decreased
4.	13	RM	CW	Recruitment	PEEP increased
5.	16	ΔP/2	CW	OD	VT decreased
6.	16	RM	No CW, CL	No Recruitment	No Change
7.	16	ΔP/2	CW	OD	No Change (pH < 7.3)
8.	16	ΔP/2	CW	OD	No Change (pH < 7.3)
9.	16	ΔP/2	CW	OD	initiate PEEP decrease
10.	14	PEEP decreased	CW	Less OD, No Derecruitment	No Change

Pat. 5	PEEP (set)	Maneuver	Finding	Interpretation	Therapeutic Measure
1.	10	RM	CW	Recruitment	PEEP increased
2.	13	ΔP/2	CW	OD	VT decreased
3.	13	RM	CW	Recruitment	PEEP increased
4.	16	ΔP/2	CW	OD	VT decreased
5.	16	ΔP/2	CW	OD	No Change (pH < 7.3)
6.	16	ΔP/2	CW	OD	No Change (pH < 7.3)
7.	16	ΔP/2	CW	OD	initiate PEEP decrease
8.	14	PEEP decreased	CW	Less OD, No Derecruitment	No Change
9.					

Pat. 6	PEEP (set)	Maneuver	Finding	Interpretation	Therapeutic Measure
1.	8	RM	CW	Recruitment	PEEP increased
2.	11	ΔP/2	No CW or CL	No relevant OD or AC	No Change
3.	11	RM	CW	Recruitment	PEEP increased
4.	14	ΔP/2	No CW or CL	No relevant OD or AC	No Change
5.	14	RM	CW	Recruitment	PEEP increased
6.	17	ΔP/2	CW	OD	VT decreased
7.	17	RM	No CW or CL	No Recruitment	No Change
8.	17	ΔP/2	No CW or CL	No relevant OD or AC	No Change
9.	17	ΔP/2	No CW or CL	No relevant OD or AC	No Change

Pat. 7	PEEP (set)	Maneuver	Finding	Interpretation	Therapeutic Measure
1.	10	RM	No CW or CL	No Recruitment	No Change
2.	10	ΔP/2	CW	OD	initiate PEEP decrease
3.	8	PEEP decreased	CW	Less OD, no derecruitment	No Change
4.	8	ΔP/2	CW	OD	VT decreased
5.	8	ΔP/2	CW	OD	initiate PEEP decrease
6.	6	PEEP decreased	CW	Less OD, no derecruitment	No Change
7.	6	ΔP/2	No CW or CL	No Relevant OD or AC	No Change
8.	6	RM	CW	Recruitment	PEEP increased
9.	9	ΔP/2	CW	OD	No Change (pH < 7.3)
10.	9	ΔP/2	No CW or CL	No Relevant OD or AC	No Change

**Pat. 8**	**PEEP (set)**	**Maneuver**	**Finding**	**Interpretation**	**Therapeutic Measure**
1.	8	RM	CW	Recruitment	PEEP increased
2.	11	ΔP/2	CW and CL	OD and AC	PEEP increased, VT decrease
3.	14	RM	No CW or CL	No Recruitment	No Change
4.	14	ΔP/2	CW	OD	VT decreased
5.	14	ΔP/2	CW	OD	No Change (pH < 7.3)
6.	14	ΔP/2	CW	OD	No Change (pH < 7.3)
7.	14	ΔP/2	CW	OD	No Change (pH < 7.3)
8.	14	RM	CW	Recruitment	PEEP increased
9.	17	ΔP/2	CW	OD	No Change (pH < 7.3)

**Pat. 9**	**PEEP (set)**	**Maneuver**	**Finding**	**Interpretation**	**Therapeutic Measure**
1.	8	RM	No CW or CL	No Recruitment	No Change
2.	8	ΔP/2	CW	OD	VT decreased
3.	8	ΔP/2	CW	OD	initiate PEEP decrease
4.	6	PEEP decreased	CL	Derecruitment	initiale RM
5.	6	RM	CW	Recruitment	PEEP increased
6.	9	ΔP/2	No CW or CL	No Relevant OD or AC	No Change
7.	9	RM	CW	Recruitment	PEEP increased
8.	12	ΔP/2	CW	OD	No Change (pH < 7.3)
9.	12	RM	CW	Recruitment	PEEP increased
10.	15	ΔP/2	CW	OD	No Change (pH < 7.3)
11.	15	RM	CW	Recruitment	PEEP increased
12.	18	ΔP/2	CW	OD	No Change (pH < 7.3)

**Pat. 10**	**PEEP (set)**	**Maneuver**	**Finding**	**Interpretation**	**Therapeutic Measure**
1.	10	RM	CW	Recruitment	PEEP increased
2.	13	ΔP/2	CW	OD	No Change (pH < 7.3)
3.	13	RM	CW	Recruitment	PEEP increased
4.	16	ΔP/2	CW	OD	No Change
5.	16	RM	No CW or CL	No Relevant OD or AC	No Change
6.	16	ΔP/2	CW	OD	No Change (pH < 7.3)
7.	16	ΔP/2	CW	OD	initiate PEEP decrease
8.	14	PEEP decreased	CW	Less OD, No Derecruitment	No Change

**Pat. 11**	**PEEP (set)**	**Maneuver**	**Finding**	**Interpretation**	**Therapeutic Measure**
1.	10	RM	CW	Recruitment	PEEP increased
2.	13	ΔP/2	CW and CL	OD and AC	PEEP increased, VT decrease
3.	16	RM	CW	Recruitment	PEEP increased
4.	19	ΔP/2	CW	OD	VT decreased
5.	19	RM	No CW or CL	No Recruitment	No Change
6.	19	ΔP/2	CW	OD	VT decreased
7.	19	ΔP/2	CW	OD	No Change (pH < 7.3)
8.	19	ΔP/2	CW	OD	initiate PEEP decrease
9.	17	PEEP decreased	CW	Less OD, No Derecruitment	No Change

Pat. 12	PEEP (set)	Maneuver	Finding	Interpretation	Therapeutic Measure
1.	12	RM	No CW or CL	No Recruitment	No Change
2.	12	ΔP/2	CW	OD	VT decreased
3. (Position Change from Supine to right lateral position)					
4.	12	ΔP/2	CW	OD	VT decreased
5.	12	ΔP/2	No CW or CL	No Relevant OD or AC	No Change
6.	12	ΔP/2	No CW or CL	No Relevant OD or AC	No Change

Pat. 13	PEEP (set)	Maneuver	Finding	Interpretation	Therapeutic Measure
1.	8	RM	CW	Recruitment	PEEP increased
2.	11	ΔP/2	CW	OD	VT decreased
3.	11	RM	CW	Recruitment	PEEP increased
4.	14	ΔP/2	No CW or CL	No Relevant OD or AC	No Change
5.	14	RM	CW	Recruitment	PEEP increased
6.	17	ΔP/2	CW	OD	No Change (PaCO2 76)
7.	17	RM	CW	Recruitment	PEEP increased
8.	20	ΔP/2	No CW or CL	No Relevant OD or AC	No Change
9.	20	RM	No CW or CL	No Recruitment	No Change
10.	20	ΔP/2	No CW or CL	No Relevant OD or AC	No Change

Pat. 14	PEEP (set)	Maneuver	Finding	Interpretation	Therapeutic Measure
1.	8	RM	CW	Recruitment	PEEP increased
2.	11	ΔP/2	CW	OD	VT decreased
3.	11	RM	CW	Recruitment	PEEP increased
4.	14	ΔP/2	CW	OD	VT decreased
5.	14	RM	No CW or CL	No Recruitment	No Change
6.	14	ΔP/2	CW	OD	No Change
7.	14	ΔP/2	CW	OD	No Change
8.	14	ΔP/2	CW	OD	No Change

Pat. 15	PEEP (set)	Maneuver	Finding	Interpretation	Therapeutic Measure
1.	8	RM	CW	Recruitment	PEEP increased
2.	11	ΔP/2	No CW or CL	No Relevant OD or AC	No Change
3.	11	RM	CW	Recruitment	PEEP increased
4.	14	ΔP/2	No CW or CL	No Relevant OD or AC	VT increased
5.	14	RM	CW	Recruitment	PEEP increased
6.	17	ΔP/2	No CW or CL	No Relevant OD or AC	No Change
7.	17	RM	No CW or CL	No Recruitment	No Change
8.	17	ΔP/2	No CW or CL	No Relevant OD or AC	VT increased
9.	17	ΔP/2	No CW or CL	No Relevant OD or AC	No Change
10.	17	ΔP/2	No CW or CL	No Relevant OD or AC	No Change

Pat. 16	PEEP (set)	Maneuver	Finding	Interpretation	Therapeutic Measure
1.	10	RM	CW	Recruitment	PEEP increased
2.	13	ΔP/2	No CW or CL	No Relevant OD or AC	VT increased
3.	13	RM	CW	Recruitment	PEEP increased
4.	16	ΔP/2	No CW or CL	No Relevant OD or AC	No Change
5.	16	RM	CW	Recruitment	PEEP increased
6.	19	ΔP/2	No CW or CL	No Relevant OD or AC	No Change
7.	19	RM	No CW or CL	No Recruitment	No Change
8.	19	ΔP/2	No CW or CL	No Relevant OD or AC	No Change

Pat. 17	PEEP (set)	Maneuver	Finding	Interpretation	Therapeutic Measure
1.	10	RM	CW	Recruitment	PEEP increased
2.	13	ΔP/2	CW	OD	VT decreased
3.	13	RM	CW	Recruitment	PEEP increased
4.	15	ΔP/2	CW	OD	VT decreased
5.	15	RM	CW	Recruitment	PEEP increased
6.	18	ΔP/2	CW	OD	No Change (pH < 7.3)

Pat. 18	PEEP (set)	Maneuver	Finding	Interpretation	Therapeutic Measure
1.	10	RM	CW	Recruitment	PEEP increased
2.	13	ΔP/2	No CW or CL	No relevant OD or AC	No Change
3.	13	RM	CW	Recruitment	PEEP increased
4.	16	ΔP/2	No CW or CL	No relevant OD or AC	No Change
5.	16	RM	No CW or CL	No Recruitment	No Change
6.	16	ΔP/2	CW	OD	VT decreased
7.	16	ΔP/2	No CW or CL	No relevant OD or AC	No Change

Pat. 19	PEEP (set)	Maneuver	Finding	Interpretation	Therapeutic Measure
1.	10	RM	CW	Recruitment	PEEP increased
2.	13	ΔP/2	No CW or CL	No relevant OD or AC	No Change
3.	13	RM	CW	Recruitment	PEEP increased
4.	16	ΔP/2	CW	OD	No Change (pH < 7.3)
5.	16	RM	No CW or CL	No Recruitment	No Change
6.	16	ΔP/2	CW	OD	No Change (pH < 7.3)

Pat. 20	PEEP (set)	Maneuver	Finding	Interpretation	Therapeutic Measure
1.	18	RM	CW	Recruitment	PEEP increased
2.	21	ΔP/2	CW and CL	OD and AC	PEEP increased, VT decrease
3.	24	ΔP/2	CW	OD	VT decreased
4.	24	ΔP/2	CW	OD	No Change (pH < 7.3)
5.	24	ΔP/2	CW	OD	initiate PEEP decrease
6.	21	PEEP decreased	CW	Less OD	No Change

Individual Patient Results

Patient	Stress (mbar)		Strain$_{release}$ (ratio)		$E_{lung,spec}$		Strain$_{recr.}$ (ratio)	
	ARDS Net	EIT	ARDS Net	EIT	ARDS Net	EIT	ARDS Net	EIT
1	12.2	14.8	3.4	0.7	3.6	20.8	0.6	0.3
2	14.4	16.2	1.7	0.8	8.5	19.7	0.6	0.7
3	9.3	7.4	0.7	1.6	13.2	4.7	0.4	0.5
4	17.5	21.6	0.8	1.1	21.9	19.6	0.6	0.4
5	15.8	14.9	1	1	15.8	15.2	0.6	0.5
6	14	22.2	0.8	2	17.5	11.2	0.6	1
7	10.8	8.4	1.1	0.9	9.8	9.5	0.6	0.4
8	10.7	16.7	0.7	1.6	15.2	10.5	0.6	0.4
9	15.2	22.7	1.2	1.6	12.7	14	0.4	0.6
10	15.2	19.7	0.8	1	18.9	19.8	0.4	0.4
11	8.5	12.4	0.5	0.8	17	14.8	0.4	0.5
12	21	18.7	1	1.2	21	16.3	0.7	0.7
13	8.8	18.4	1.1	1.5	8	12.6	0.7	0.5
14	17.5	20.8	0.8	1.1	21.9	19.8	0.5	0.4
15	11.7	15.9	0.6	1.8	19.5	8.7	0.5	0.7
16	11.4	16.7	0.5	1	22.8	17.5	0.4	0.5
17	12.2	15.8	0.7	1.4	17.5	11.2	0.4	0.3
18	21.2	24.8	0.8	1.5	26.6	16.2	0.7	0.8
19	14.3	15.8	0.8	1.1	17.9	14.9	0.7	0.7
20	19.5	19.2	2.2	3.4	8.9	5.7	1	0.6

Table S1. Stress and Strain in individual patients after mechanical ventilation according to the ARDS Network protocol low positive end-expiratory pressure (PEEP) table (ARDSNet) and after four hours of mechanical ventilation according to the electrical impedance tomography (EIT) based protocol. Strain$_{release}$ = release-derived strain, calculated as volume change above release-derived functional residual capacity (FRC$_{rel}$), normalized to FRC$_{rel}$; Strain$_{recr.}$ = recruitment-adjusted strain, calculated as volume change above recruitment-adjusted FRC (FRC$_{recr.}$), normalized to FRC$_{recr}$; $E_{lung,spec}$ = specific lung elastance.

Patient	PaO_2/FiO_2 (mmHg)		$PaCO_2$ (mmHg)		pH	
	ARDS Net	EIT	ARDS Net	EIT	ARDS Net	EIT
1	137	130	48	55	7.24	7.24
2	148	203	48	53	7.42	7.37
3	120	234	46	78	7.48	7.28
4	132	283	51	50	7.33	7.33
5	148	253	58	60	7.3	7.27
6	189	333	72	62	7.25	7.33
7	162	148	64	66	7.3	7.27
8	215	268	54	73	7.39	7.26
9	168	170	53	71	7.32	7.2
10	152	165	59	58	7.24	7.24
11	194	182	43	58	7.39	7.26
12	135	191	56	52	7.27	7.26
13	138	227	68	68	7.36	7.34
14	138	220	52	63	7.36	7.29
15	182	172	43	45	7.29	7.27
16	190	244	81	76	7.33	7.35
17	127	133	58	63	7.31	7.27
18	105	163	76	59	7.24	7.36
19	145	218	58	51	7.18	7.24
20	96	250	73	59	7.26	7.33

Table S2. Gas exchange in individual pateints after mechanical ventilation according to the ARDS Network protocol low positive end-expiratory pressure (PEEP) table (ARDSNet) and after four hours of mechanical ventilation according to the electrical impedance tomography based protocol (EIT).

Patient	V_T (ml/kg PBW)		$P_{aw.plat}$ (mbar)		PEEP (mbar)		C_{rs} (ml/mbar)	
	ARDS Net	EIT	ARDS Net	EIT	ARDS Net	EIT	ARDS Net	EIT
1	5.3	6.4	19.4	22.5	10	12	31.7	33.4
2	6.2	7	21.1	28.2	8	17	34.3	41.6
3	5.9	4.5	20.6	22.1	8	14	41.8	43.2
4	6.8	5.5	21.4	26.9	7	14	29.6	24.7
5	5.7	5.3	20.9	23.2	10	14	30.1	33.6
6	4.6	6.7	23.2	23.3	8	17	39	57.4
7	6.1	5.2	20.4	19.2	10	9	47.1	37.7
8	5.7	4.3	18.6	29.4	8	17	36.8	22.8
9	5.8	5.3	27.7	32.1	8	18	37.2	42.6
10	5.7	6.3	20.2	25.2	10	14	34.9	35.5
11	5.6	5.6	20.1	26.9	10	17	50.9	53.3
12	6.1	5.6	26.1	29.4	12	15	23.6	20.8
13	5.8	6	15.8	29	8	20	57.3	51.1
14	5.3	3.4	24.1	27.9	8	14	29.9	21.7
15	5.3	6.8	15.5	26.5	8	17	38.4	37.9
16	5.5	6.3	20.1	29.6	10	19	41.1	41.4
17	6.5	5.5	22.9	31	10	18	39.8	30.5
18	5.5	6.7	23.1	29.9	10	16	38.6	41.3
19	5.8	6.3	27.7	32.2	10	16	28.3	28.3
20	5.9	6.3	27.5	28.9	18	21	54.3	58.9

Table S3. Ventilator parameters in individual pateints after mechanical ventilation according to the ARDS Network protocol low positive end-expiratory pressure (PEEP) table (ARDSNet) and after four hours of mechanical ventilation according to the electrical impedance tomography based protocol (EIT). V_T = tidal volume, PBW = predicted pody weight, $P_{aw,plat}$ = plateau airway pressure, PEEP = positive end-expiratory pressure, C_{rs} = respiratory system compliance.

Patient	V_{PEEP} (ml)		C_{lung} (ml/mbar)		ΔP_{aw} (mbar)		ΔP_{TP} (mbar)	
	ARDS Net	EIT	ARDS Net	EIT	ARDS Net	EIT	ARDS Net	EIT
1	317	400.8	49.8	50.4	8.8	10.1	5.6	6.7
2	274.4	707.2	50.1	72.7	11.4	10.3	7.6	5.9
3	334.4	604.8	83.6	127.9	10	7.6	5	2.5
4	207.2	345.8	36.1	31.6	13.8	13.1	11.3	10.3
5	301	470.4	39.9	52.3	10.6	8.9	8	5.7
6	312	975.8	49.6	69.3	9.4	9.4	7.4	7.7
7	471	339.3	89.4	85.8	9.4	9.7	4.8	4.3
8	294.4	387.6	64.3	40.7	10.2	12	5.9	6.9
9	297.6	766.8	45.5	52.6	13.8	8.9	8.5	7.2
10	349	497	46.6	45.5	9.8	11	7.5	8.5
11	509	906.1	115.2	114.8	8.8	8.5	3.9	3.9
12	283.2	312	29.5	32.7	13.3	13.5	10.4	8.6
13	458.4	1022	102.6	80.8	6.9	7.9	3.8	5
14	239.2	303.8	38.9	28.5	14.1	12.5	10.9	9.5
15	307.2	644.3	49.5	62.2	6.7	8.6	5.2	5.3
16	411	745.2	70.9	72.1	8.8	10.1	5.1	5.8
17	398	549	72.3	59.6	10.8	12.2	5.9	6.1
18	386	660.8	40.9	47.6	10.7	11.7	10.1	10.5
19	283	452.8	49.9	54.5	12.6	13.7	7.2	7.1
20	976.8	1178	76.7	89	8.2	8	5.8	5.3

Table S4. PEEP volume (V_{PEEP}), lung Compliance (C_{lung}), airway driving pressure (ΔP_{aw}), and transpulmonary driving pressure (ΔP_{TP}) in individual patients after mechanical ventilation according to the ARDS Network protocol low positive end-expiratory pressure (PEEP) table (ARDSNet) and after four hours of mechanical ventilation according to the electrical impedance tomography based protocol (EIT).

Patient	SD_{RVD} (%)		$P_{tp,plat}$ (mbar)		$P_{tp,exp}$ (mbar)		Tidal Power (J/min)	
	ARDS Net	EIT	ARDS Net	EIT	ARDS Net	EIT	ARDS Net	EIT
1	7	7	6.2	9.8	0.6	3.1	3.6	3
2	7	4	1.8	10.5	-5.8	4.6	5.1	3.9
3	11	4	-1.7	6.6	-6.7	4.1	4.9	2.6
4	7	8	7.1	10.1	-4.2	-0.2	6.1	5.8
5	12	10	3	4.2	-5	-1.5	4.5	3.1
6	4	5	1.3	8.3	-6.1	0.6	3.7	4.4
7	6	7	6	3.3	1.2	-1	4.1	3.5
8	12	5	7.3	11.6	1.4	4.7	3.8	4.6
9	8	8	2.4	10.6	-6.1	3.4	6.2	5.4
10	8	8	6.2	10.7	-1.3	2.2	4	4.6
11	8	8	1.2	1.9	-2.7	-2	3.9	4.5
12	13	7	4.5	7.8	-5.9	-0.8	6.6	6.5
13	6	5	5.3	12.5	1.5	7.5	2.6	3.3
14	12	5	9.4	13.9	-1.5	4.4	6.4	5.8
15	4	4	0	7.1	-5.2	1.8	2	3
16	7	5	-2.3	13.5	-7.4	7.7	3.4	4.1
17	10	6	5.9	10.1	0	4	5.9	6.5
18	4	7	4.2	6.6	-5.9	-3.9	5.2	5.2
19	10	8	11	12	3.8	4.9	5.3	5.2
20	9	10	9.3	11	3.5	5.7	3.9	4.1

Table S5. Standard deviation of regional ventilation delay (SD_{RVD}), transpulmonary plateau pressure ($P_{TP,plat}$), end-expiratory transpulmoary pressure ($P_{TP,exp}$) and tidal power in individual patients after mechanical ventilation according to the ARDS Network protocol low positive end-expiratory pressure (PEEP) table (ARDSNet) and after four hours of mechanical ventilation according to the electrical impedance tomography based protocol (EIT).

Patient	EELV (ml)		$FRC_{release}$ (ml)		$FRC_{recr.}$ (ml)	
	ARDS Net	EIT	ARDS Net	EIT	ARDS Net	EIT
1	1359.5	2890.7	372.6	1890	1029.7	2482.7
2	1578.7	2330.5	720.5	1516	1239.3	1585.1
3	2114.1	2365	1496.6	1037	1758.7	1755
4	1380.2	2039.5	1009	1125	1155.4	1679.1
5	1447.2	2127.3	875.5	1228	1137.3	1646.9
6	1456.5	2460.6	994.9	1006	1128.7	1466.6
7	2054.3	2085.4	1209.8	1298	1521.7	1727.9
8	1479	1961.9	1093.4	867.7	1173.4	1554.6
9	2123	2857.6	1164.7	1237	1817.7	2040.5
10	2263.1	2809.6	1422.1	1605	1906.7	2306.9
11	2905.1	4067.5	2193.4	2456	2376.8	3098.1
12	1182.2	1267.5	757.2	722	870.2	936.2
13	1881.7	3988.4	1107.5	1788	1365.8	2904.8
14	1549.8	1920.8	1098.4	1069	1293.1	1597.9
15	1474.9	2032.2	1058.4	833.9	1152.2	1365.1
16	2375.4	3304.5	1823.1	1908	1927.3	2521.9
17	2927.8	3585.1	2031.6	1632	2469.6	3019
18	1695.1	2181.6	1141.9	1059	1240.1	1476
19	1417.8	1780.9	999	1053	1061.3	1306.1
20	2622	3927.4	959.2	1010	1569.2	2689.7

Table S6. End-expiratory lung volume (EELV), release-derived functional residual capacity ($FRC_{release}$) and recruitment-adjusted functional residual capacity (FRC_{recr}) in individual patients after mechanical ventilation according to the ARDS Network protocol low positive end-expiratory pressure (PEEP) table (ARDSNet) and after four hours of mechanical ventilation according to the electrical impedance tomography based protocol (EIT).

Patient Example with EIT Screenshots (Patient 4)

1. PEEP 7 → RM → PEEP 10

A recruitment maneuver (RM) was started at a PEEP of 7 mbar (A:7), increasing PEEP to 10 mbar immediately after RM (B:10). A substantial compliance win (CW) was detected in comparison to A:7, indicating recruitment. Therefore, the higher PEEP level of 10 mbar was kept.

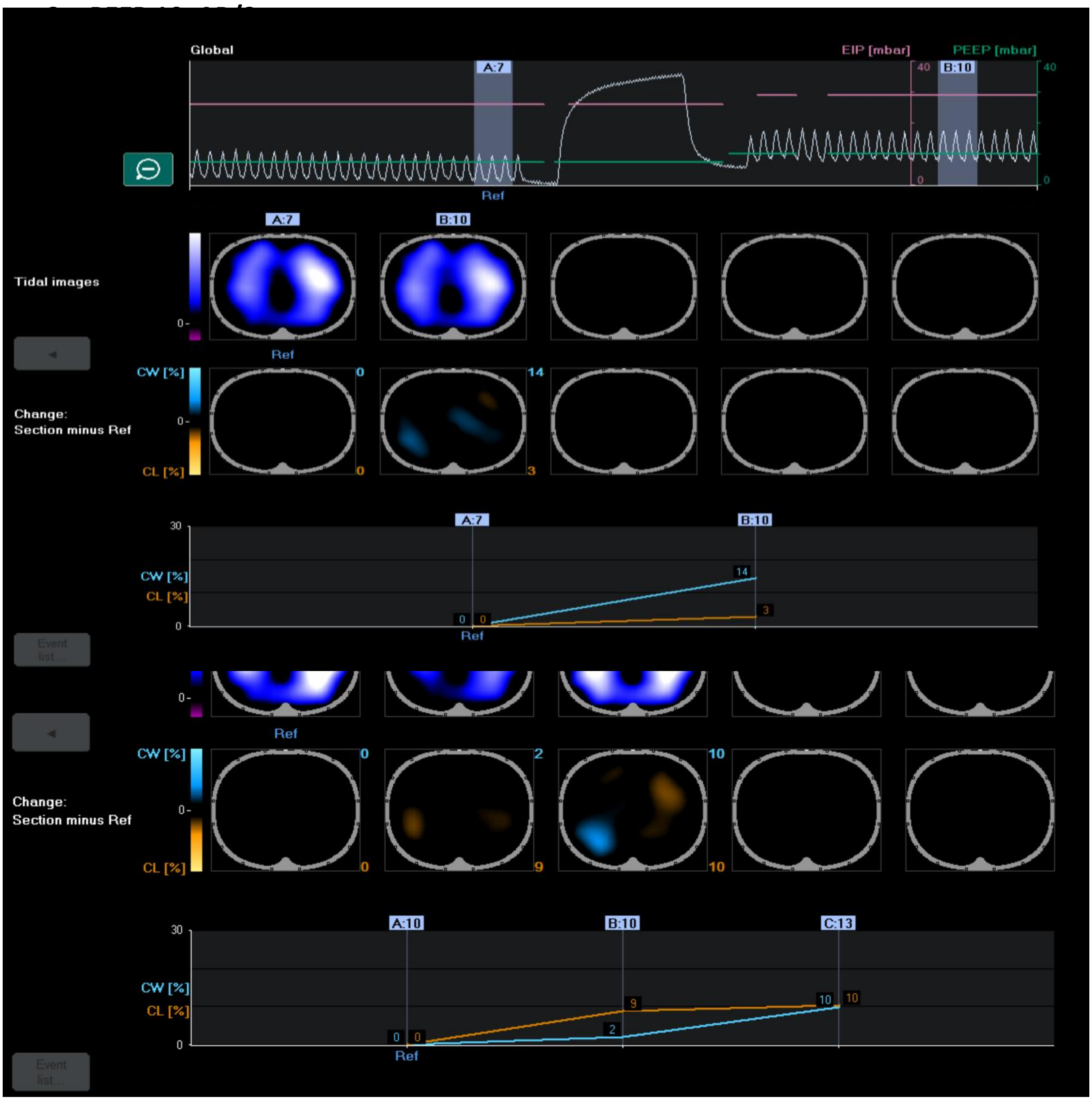

3. PEEP 13: ΔP/2

While ventilating the patient at a PEEP level of 13 mbar (A:13), a reduction in driving pressure by 50% (ΔP/2) was performed (B:13). Substantial Compliance Win (CW) was detected with lower ΔP in comparison to A:13. According to the protocol, this was interpreted as possible overdistension and tidal volume was subsequently reduced by 1 ml/kg PBW to alleviate overdistension (C:13).

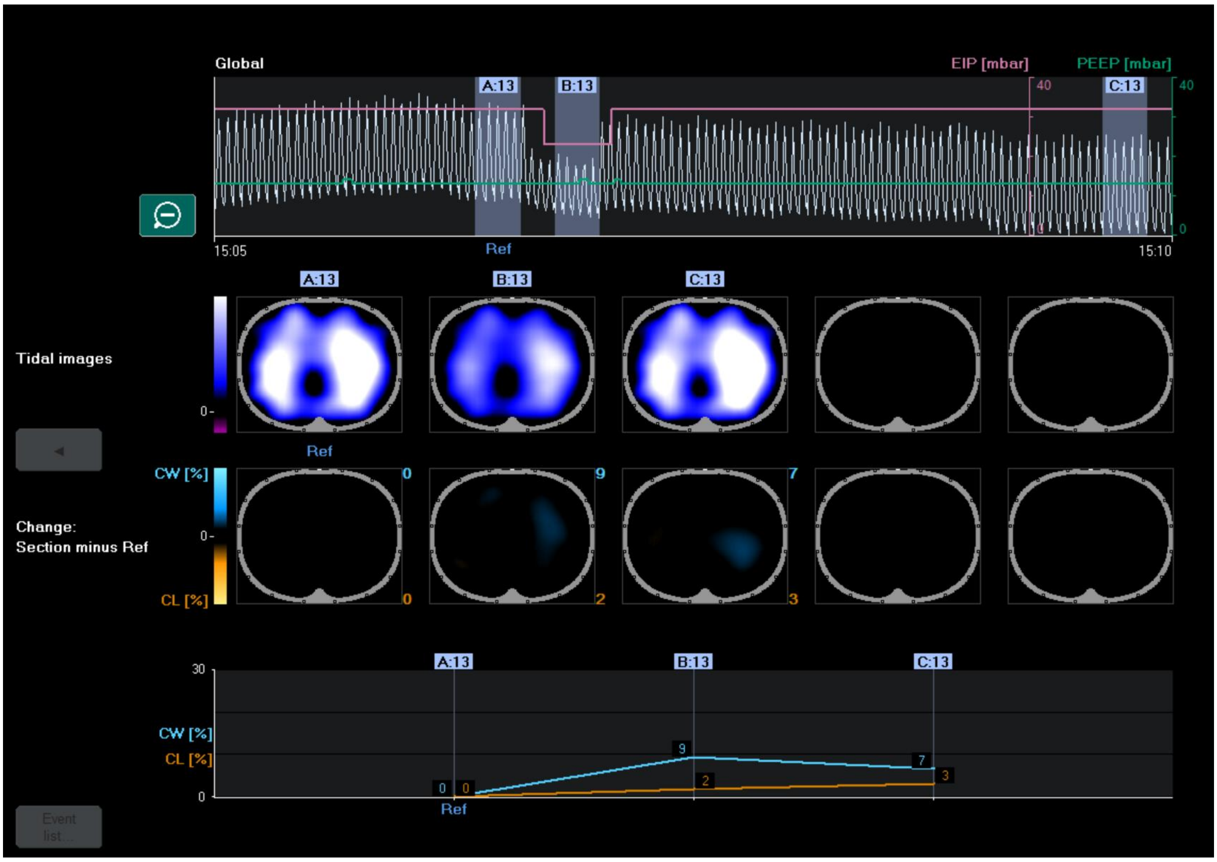

4. PEEP 13 → RM → PEEP 16

Another recruitment maneuver (RM) was started at a PEEP of 13 mbar (A:13), increasing PEEP to 16 mbar immediately after RM (B:16). A substantial compliance win (CW) was detected in comparison to A:13. According to the protocol, this was interpreted as recruitment. Therefore, the higher PEEP level of 16 mbar was kept.

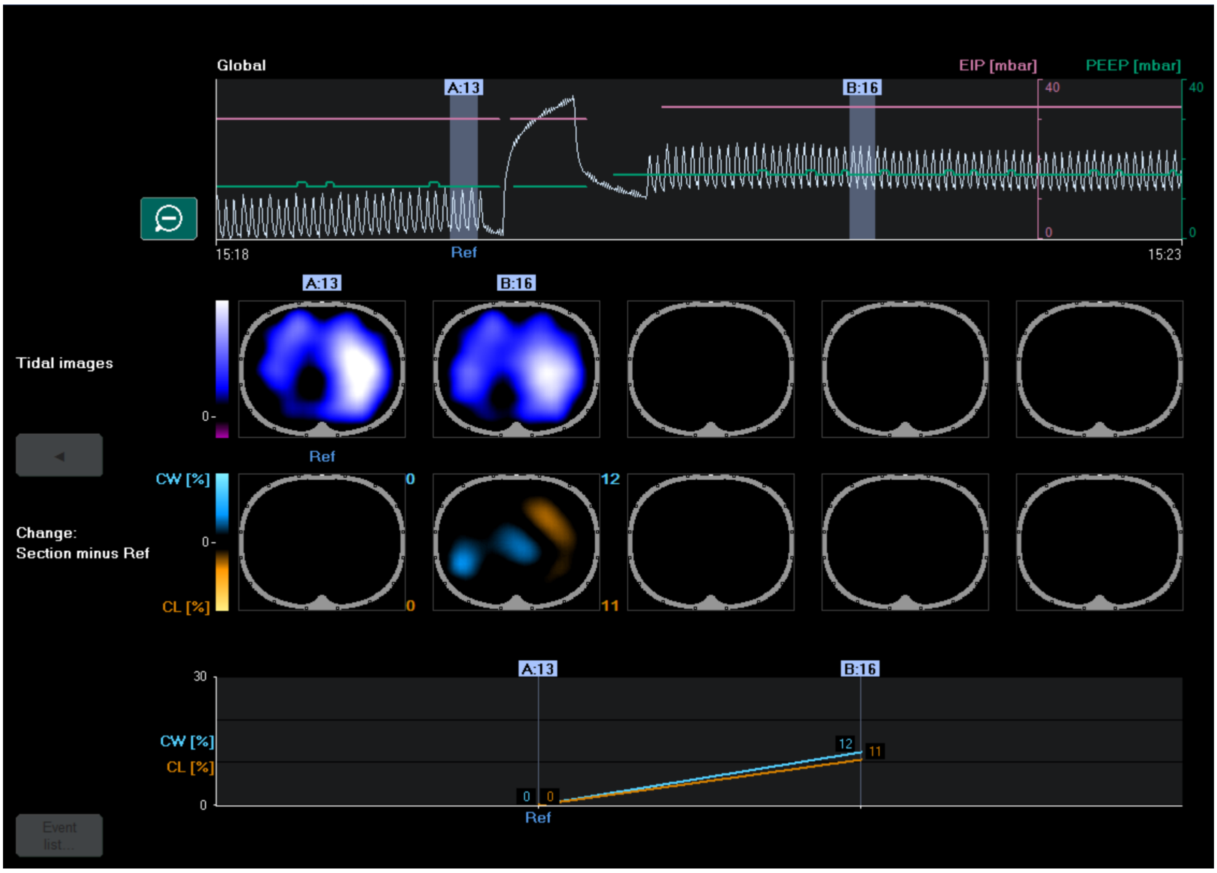

5. PEEP 16: ΔP/2

While ventilating the patient at a PEEP level of 16 mbar (A:16), a reduction in driving pressure by 50% (ΔP/2) was performed (B:16). Substantial Compliance Win (CW) was detected with lower ΔP in comparison to A:16. According to the protocol, this was interpreted as possible overdistension and VT was reduced by another 0.5 ml/kg.

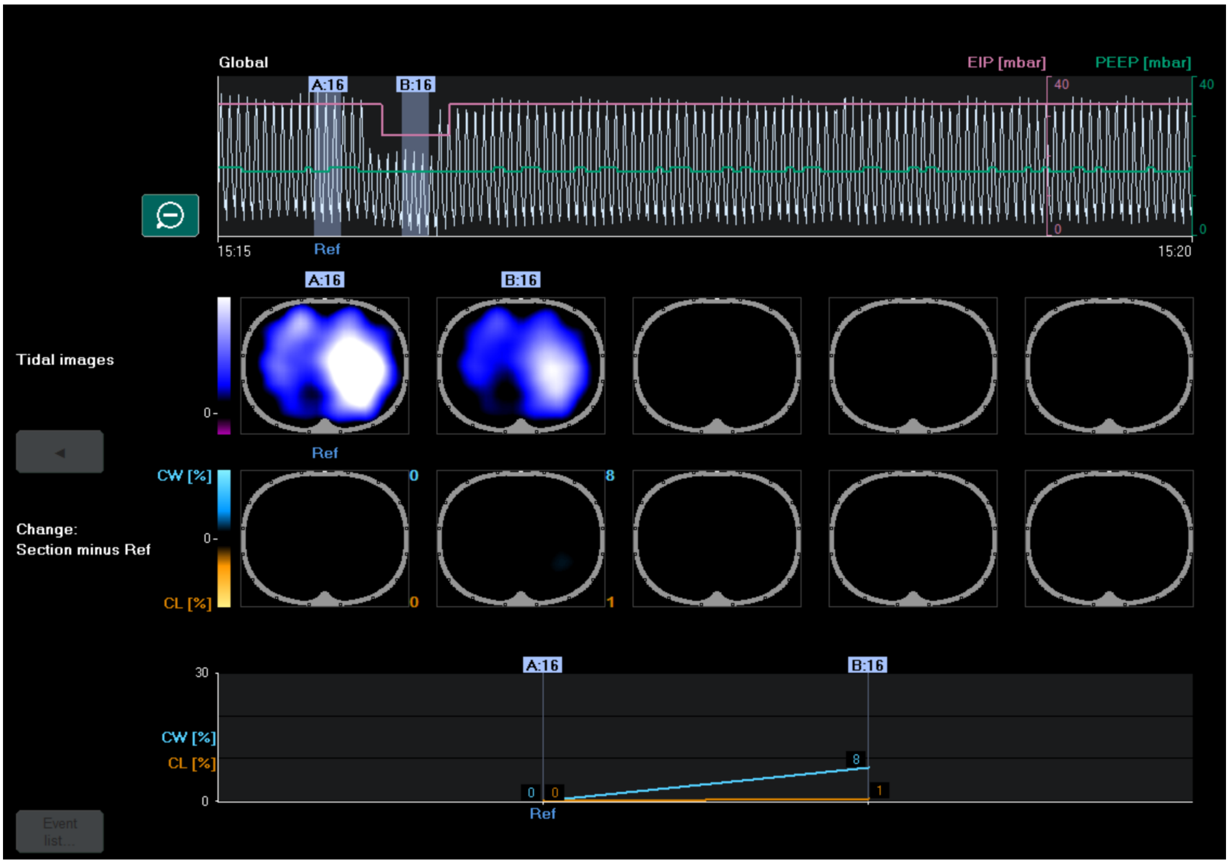

6. PEEP 16 → RM → PEEP 19

Another recruitment maneuver (RM) was started at a PEEP of 16 mbar (A:16), increasing PEEP to 19 mbar immediately after RM (B:19). A substantial compliance loss (CL) was detected in comparison to A:16 with no relevant compliance win (CW). According to the protocol, this was interpreted as absence of recruitment and presence of overdistension with the higher PEEP level. Therefore, the PEEP level was reduced back to the previous level of 16 mbar.

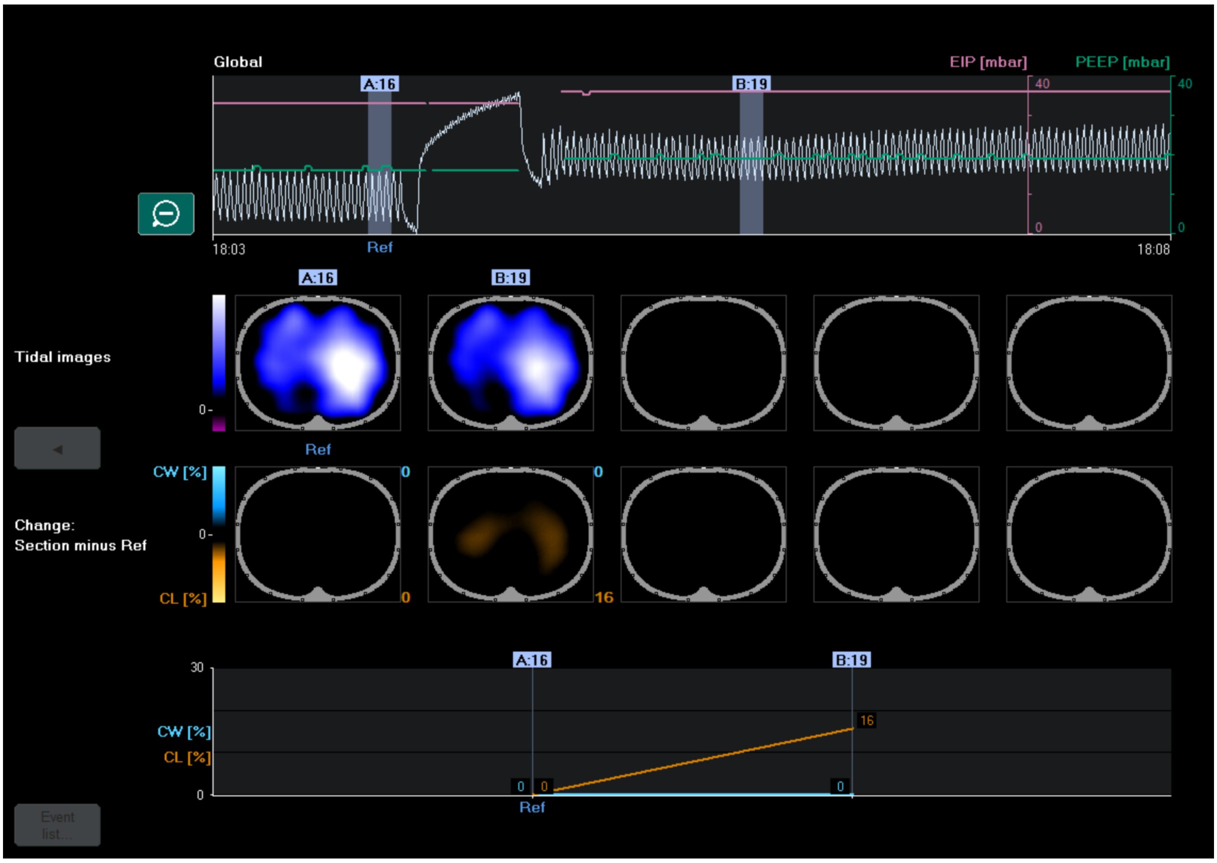

7. PEEP 16: ΔP/2

While ventilating the patient at a PEEP level of 16 mbar (A:16), a reduction in driving pressure by 50% (ΔP/2) was performed (B:16). Substantial Compliance Win (CW) was detected with lower ΔP in comparison to A:16. According to the protocol, this was interpreted as possible overdistension but due to respiratory acidosis (pH 7.28), VT could not be reduced any further. The last protocol-driven PEEP increase was less than two hours ago, therefore, PEEP was not decreased at this point and ΔP and PEEP were kept constant.

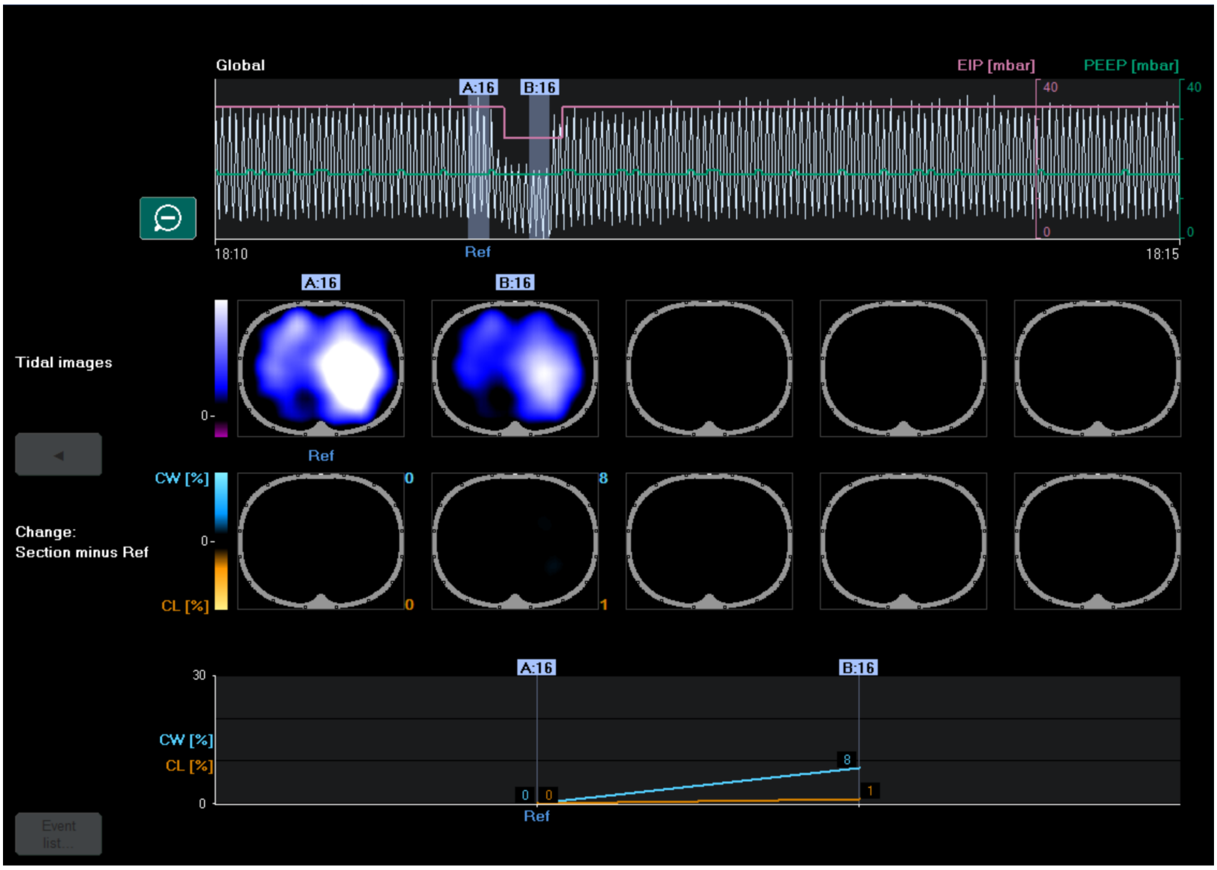

8. PEEP 16: ΔP/2

While ventilating the patient at a PEEP level of 16 mbar (A:16), a reduction in driving pressure by 50% (ΔP/2) was performed (B:16). Substantial Compliance Win (CW) was detected with lower ΔP in comparison to A:16. According to the protocol, this was interpreted as possible overdistension but due to respiratory acidosis (pH 7.26), VT could not be reduced any further. The last protocol-driven PEEP increase was less than two hours ago, therefore, PEEP was not decreased at this point and ΔP and PEEP were kept constant.

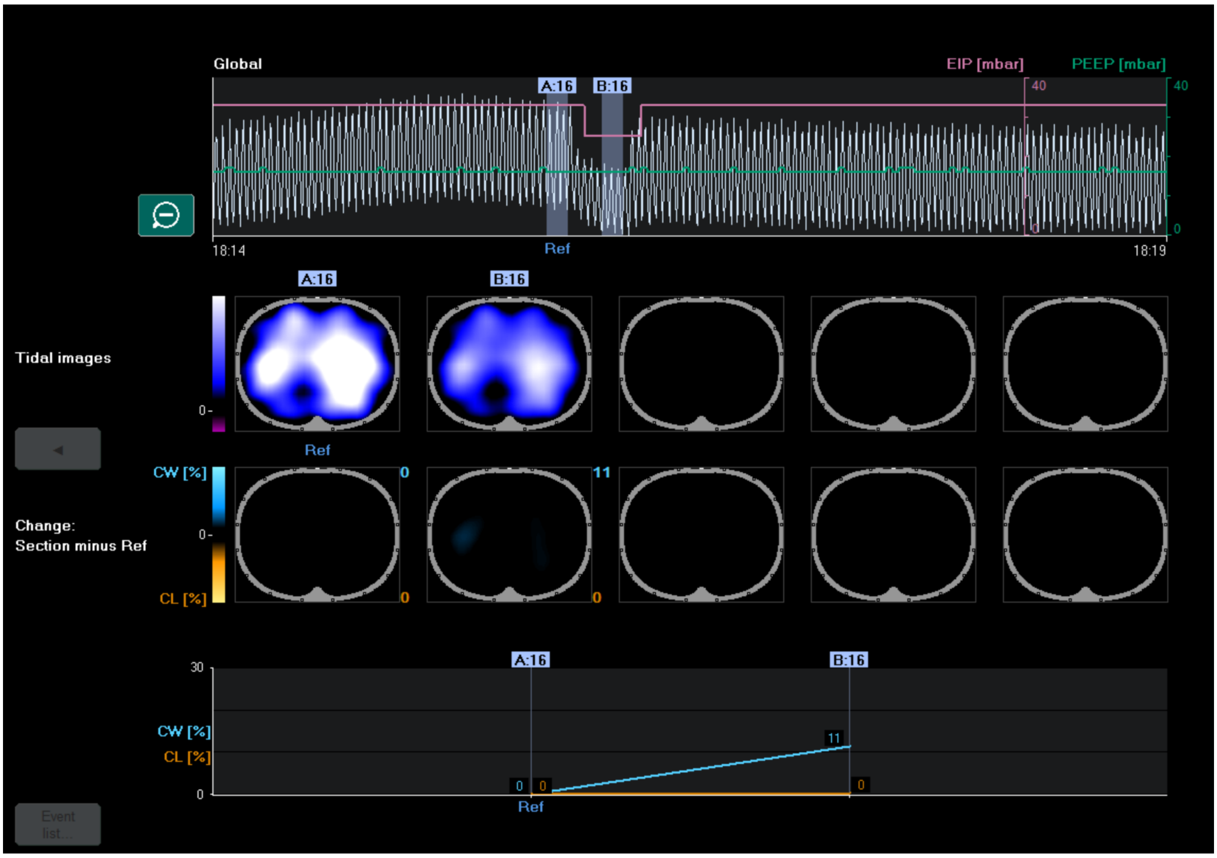

9. PEEP 16: ΔP/2

While ventilating the patient at a PEEP level of 16 mbar (A:16), a reduction in driving pressure by 50% (ΔP/2) was performed (B:16). Substantial Compliance Win (CW) was detected with lower ΔP in comparison to A:16. According to the protocol, this was interpreted as possible overdistension. The last protocol-driven PEEP increase was more than two hours ago, therefore, PEEP was now decreased by 2 mbar (see 10.: PEEP-decrease).

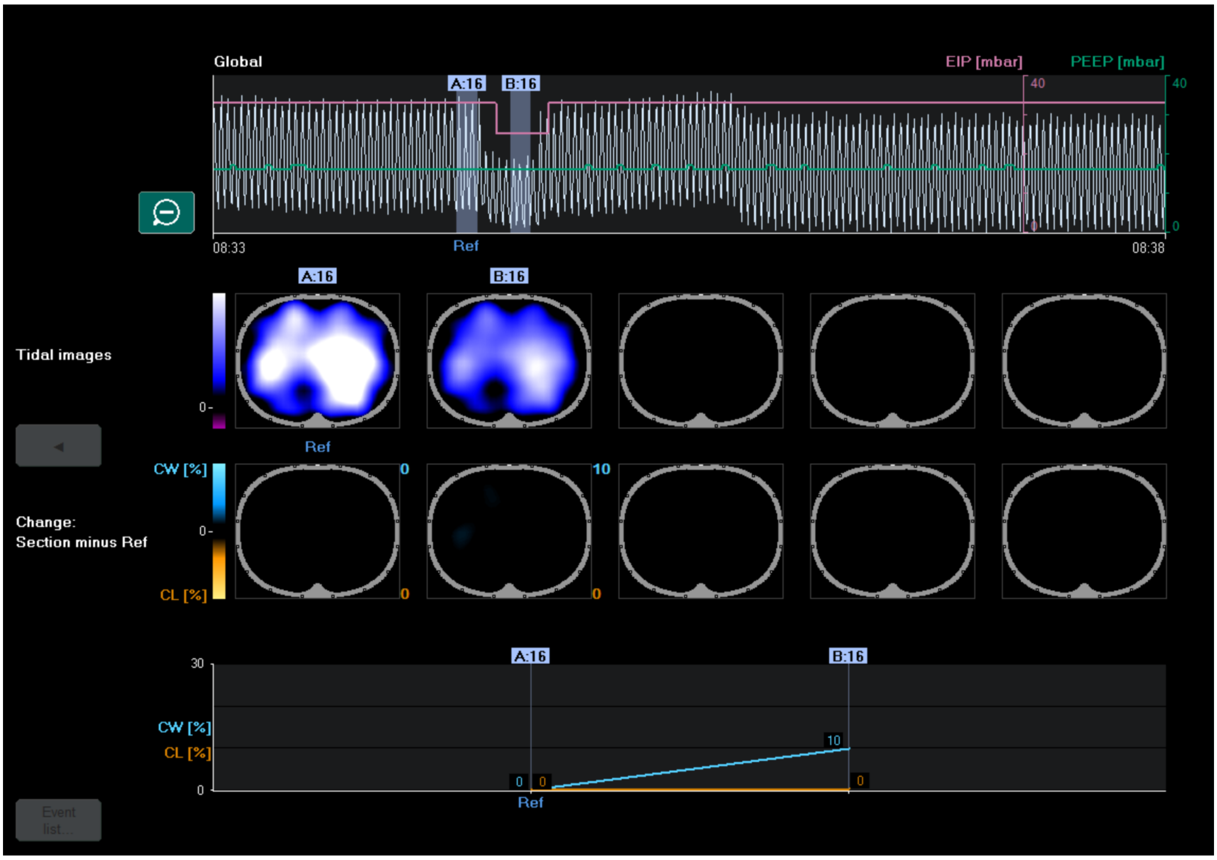

10. PEEP decrease 16 → 14

A PEEP decrease was initiated, reducing the PEEP level from 16 (A:16) to 14 (B:14) mbar. Substantial Compliance Win (CW) was detected, which, according to the protocol, was interpreted as alleviation of previous overdistension. Simultaneously, no relevant compliance loss (CL) was detected, which was interpreted as absence of derecruitment following PEEP decrease. Therefore, the lower PEEP level was kept.

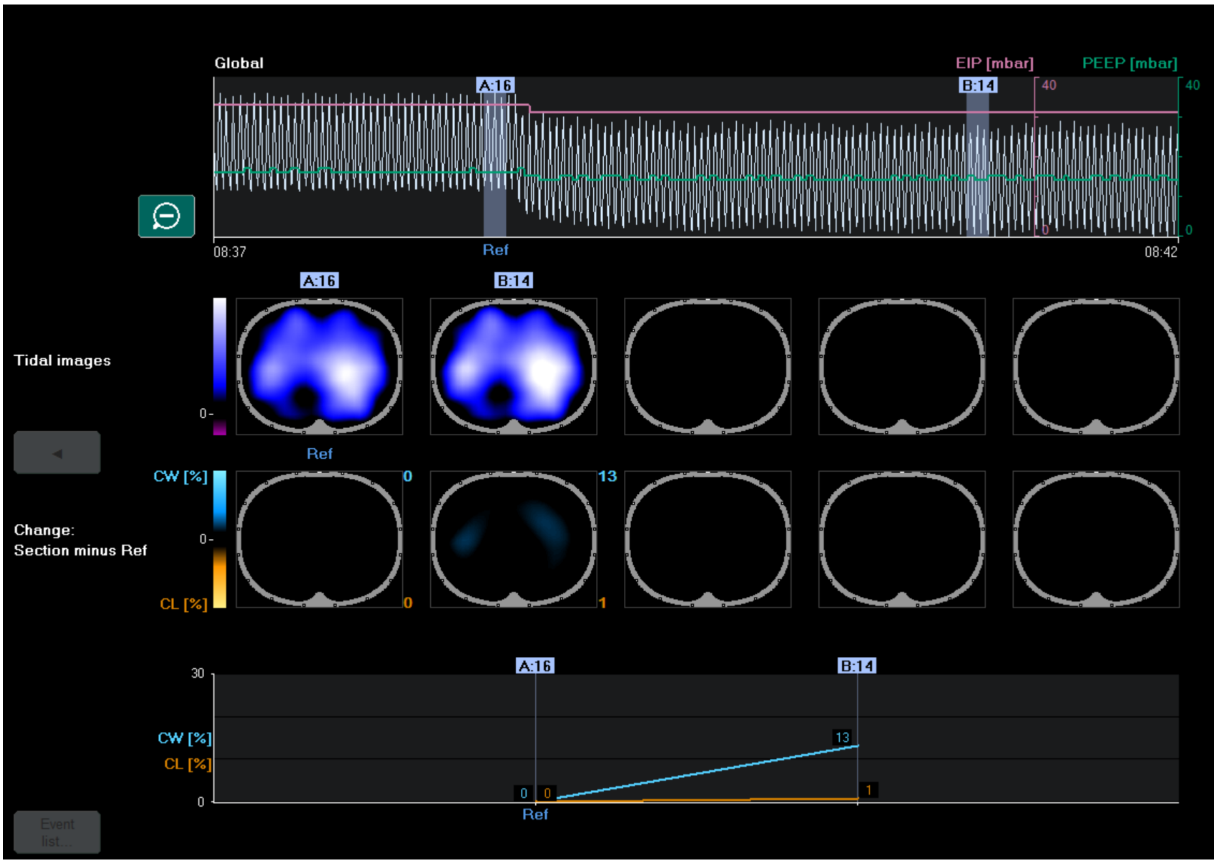

Supplement: Supplementary file 1 — Additional file 1. Protocol for adjustment of VT and PEEP with EIT, treatment courses of individual patients during EIT-based adjustment, individual patient results and patient example with EIT screenshots [file 13613_2021_877_MOESM1_ESM.pdf]
